# Thrombolytic therapy versus cardiac surgery for left-sided prosthetic heart valve thrombosis: A systematic review and meta-analysis

**DOI:** 10.1016/j.ahjo.2026.100849

**Published:** 2026-07-29

**Authors:** Babak Bagheri, Sahar Eskandari, Saeid Soltani, Rozita Jalalian, Jamshid Yazdani Charati, Masoud Moradi, Mohammadreza Iranian

**Affiliations:** a Department of Cardiology, Faculty of Medicine, Cardiovascular Research Center, Mazandaran University of Medical Sciences, Sari, Iran; b Department of Biostatistics, Faculty of Health, Mazandaran University of Medical Sciences, Sari, Iran; c Rajaie Cardiovascular Medical and Research Center, School of Medicine, Iran University of Medical Sciences, Tehran, Iran

**Keywords:** Prosthetic valve thrombosis, Thrombolytic therapy, Fibrinolytic, Heart valve surgery, Meta-analysis

## Abstract

**Introduction:**

The optimal treatment of prosthetic heart valve thrombosis (PVT) remains vigorously debated in international guidelines. The present study was designed to compare the efficacy and safety of thrombolytic therapy and surgery in left-sided PVT, incorporating recent data to inform clinical decision-making.

**Methods:**

This study is a systematic review with meta-analysis. We searched PubMed, Scopus, and Embase up to Jun 30, 2025. Eligible studies were those reporting treatment strategies and outcomes of patients with left-sided PVT. The primary outcome was treatment success (survival for surgery; complete or partial improvement of valve function and hemodynamic success not requiring surgery after thrombolysis).

**Results:**

114 studies were included, representing 6514 patients with 6744 PVT episodes. The success rate in both treatment groups was the same: 83.9% (95% CI: 82.2–85.4; I^2^ = 19.8%) for thrombolysis versus 85.1% (95% CI: 83.3–86.7; I^2^ = 27%) for surgery (*P*-value = 0.607). The mortality rate was lower with thrombolysis (8.5%; 95% CI: 7.5–9.8; I^2^ = 0%) than surgery (15%; 95% CI: 13.4–16.8; I^2^ = 26.3%) (P-value = 0.001). Other secondary outcomes showed no significant differences. In the slow/very slow thrombolytic infusion subgroup analysis, the success rate was 91.4% (95% CI: 87–94; I^2^ = 44.9%) and the mortality was 2.1% (95% CI: 1.2–3.3; I^2^ = 0%).

**Conclusions:**

This systematic review suggests that fibrinolytics are a reasonable option alongside surgery for left-sided PVT, with similar success rates and lower mortality. However, due to observational evidence, results should be interpreted cautiously and randomized controlled trials (RCTs) are needed.

## Highlights

• The optimal treatment of prosthetic heart valve thrombosis (PVT) remains vigorously debated in international guidelines.
• We tried to compare the efficacy and safety of thrombolytic therapy and surgery in left-sided PVT.
• This systematic review and meta-analysis showed that fibrinolytics are a suitable treatment option alongside surgery for management of prosthetic heart valve thrombosis.

## 1 Introduction

Prosthetic heart valve thrombosis (PVT) is a rare but severe complication, with an annual incidence of 0.1% to 5.7% for mechanical valves. It is characterized by thrombus formation on or near the prosthesis, causing dysfunction or obstruction. Clinical presentation ranges from asymptomatic to acute dyspnea, embolism, or cardiogenic shock. Risk is highest within 3 months post-implantation, with certain valve types, and in mitral or tricuspid positions compared to aortic position [1], [2].

Treatment options include cardiac surgery (valve replacement or thrombectomy) and systemic thrombolysis. The optimal treatment of PVT remains vigorously debated in international guidelines. European guidelines recommend urgent or emergency valve replacement, whereas the American guidelines favor of systemic low-dose slow infusion of a thrombolytic agent [2], [3].

This systematic review and meta-analysis was designed to compare the efficacy and safety of thrombolytic therapy and surgery in left-sided PVT, incorporating recent data to inform clinical decision-making.

## 2 Methods

### 2.1 Search strategy

Per PRISMA guideline [4], we searched PubMed, Scopus, and Embase up to Jun 30, 2025, using terms: prosthetic heart valve, mechanical heart valve, thrombosis, prosthetic heart valve thrombosis, prosthetic valve thrombosis, surgery, valve replacement, thrombectomy, fibrinolytic, thrombolytic, streptokinase, urokinase, tissue plasminogen activator, tenecteplase, alteplase, reteplase. For the purpose of this analysis, eligible studies were those reporting treatment strategies and outcomes of patients with left-sided PVT. Eligible studies were required to meet all of the following inclusion criteria:

1. Reported data on treatment strategies for left-sided PVT, including thrombolytic therapy or surgery, with more than 8 patients.
2. Report data on at least success rate and in-hospital death.

The bibliographic records retrieved were downloaded, imported, and deduplicated using EndNote software version X7 (Clarivate Analytics, Philadelphia, PA.). Repetitive studies, case series (<8 patients) or case reports, studies that did not provide data on patient treatments or where survival outcomes were unknown, studies limited to right-sided valves (tricuspid/pulmonary) and studies on bio prosthetic valves were excluded. Further details regarding the search strategy used for each database are provided in supplement data 1 (Table S1).

### 2.2 Outcome measures

The primary outcome was treatment success (survival for surgery; complete or partial improvement of valve function and hemodynamic success not requiring surgery after thrombolysis). It is important to note that the definition of success varied across studies, typically including restoration of normal transvalvular gradients, improvement in NYHA functional class, and survival for surgery, but with some degree of heterogeneity in exact criteria.

Secondary outcomes were in-hospital mortality, nonfatal stroke and transient ischemic attack (TIA), non-central nervous system (CNS) embolism, nonfatal intracranial hemorrhage (ICH) and nonfatal major bleeding, as defined by study authors.

### 2.3 Data collection and quality assessment

Two reviewers independently screened titles and abstracts of the articles based on the inclusion and exclusion criteria. The eligible studies were evaluated by reviewing the full texts. Conflicts were discussed with a third reviewer and resolved by mutual agreement. The risk of bias assessment was evaluated, and the quality of the articles was assessed using study quality assessment tools suggested by the National Heart, Lung and Blood Institute (NHLBI) [5]. The studies were assessed and divided into overall rating qualities of good, fair, and poor. The risk of bias assessment of included studies is presented in supplement data 1 (Table S2).

### 2.4 Statistical analysis

To perform the meta-analysis, we estimate pooled outcome proportions in one group at one time-point, by a random effects model. Unlike direct comparison meta-analysis, all study results were pooled and then separated into the two treatment strategy subgroups: studies evaluating surgery and those evaluating thrombolytic therapy. Pooled subgroup results were compared by random effects. These values are computed within subgroups and the difference between them was considered statistically significant if *P*-value <0.05. The results were also presented by forest plots. It should be noted that comparisons between the thrombolysis and surgery groups are based on pooled single-arm proportions and are not adjusted for baseline differences between treatment groups due to the lack of individual patient data.

To assess the between-study heterogeneity, Higgin's I^2^ and Cochran's Q values were calculated. The I^2^ > 25% was considered as high between-study heterogeneity. Sensitivity analyses also were performed to find the potential source of the between-study heterogeneity, using the leave-one-out method. To assess the publication bias, studies were presented in a funnel plot and Peter's linear regression and Egger's regression tests were performed. Figures and tables in Supplementary data 2 that lists the studies excluded at each step of the analysis due to high heterogeneity or high influence on publication bias.

All analyses were performed using RStudio software version 1.2.5042, using “metagen” and “metafor” packages. Details about analysis is reserved by the authors and will be provided by the corresponding author upon reasonable request.

## 3 Results

### 3.1 Study selection and the characteristics of the included studies

**Fig. 1:**
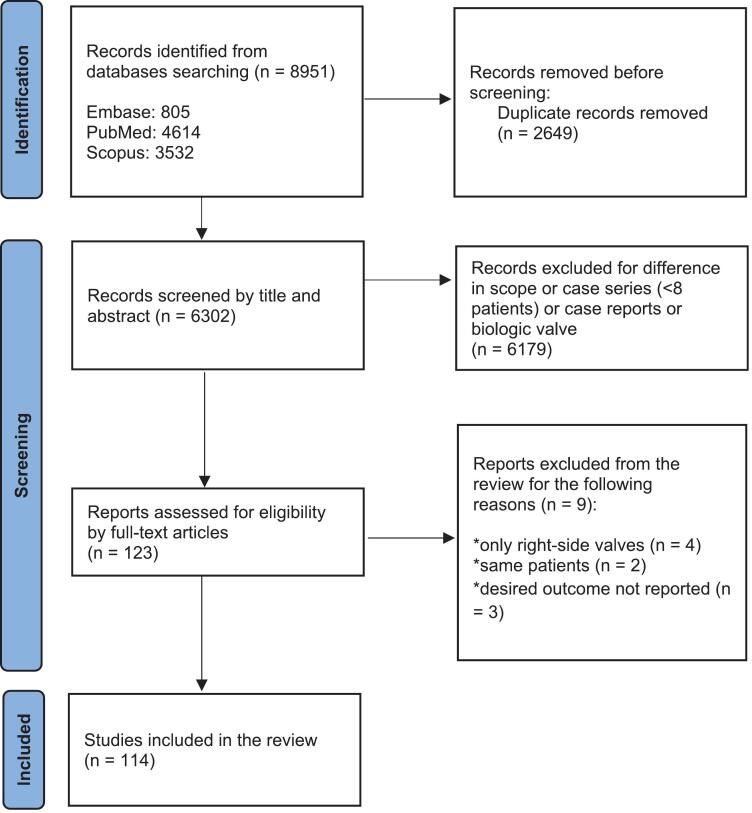
Preferred Reporting Items for Systematic Reviews and Meta-analyses (PRISMA) flowchart.

Fig. 1 shows the process of selecting studies. Of 8951 records, 114 studies were included, representing 6514 patients with 6744 PVT episodes. 112 studies were observational and only two studies were randomized clinical trial (RCT), one of which compared surgery with fibrinolytic therapy. Based on the NHLBI article quality assessment tools, 20 articles were of good quality and the rest were of fair quality. 4195 episodes (62%; 85 studies) were treated with thrombolysis and 2549 episodes (38%; 46 studies) with surgery. The mitral valve position was the most common site for thrombosis (75%). In the thrombolysis group, 38.6% of patients and in the surgery group, 30% of patients were male. Rates of NYHA class IV was 25% in thrombolysis group (41 studies reported NYHA class) and 38% in surgery group (37 studies reported NYHA class). Streptokinase (58.8%) and alteplase (27.5%) were common thrombolytic. 20% of thrombolysis group (11 studies, 1021 episodes) used slow/very slow alteplase infusion. The terms slow infusion (6 h) or very slow infusion (25 h) refer to specific protocols for administering thrombolytic drugs (as opposed to the traditional, rapid administration over 60 to 90 min) that have recently been developed to reduce the risk of complications (such as bleeding) while maintaining efficacy. The principal characteristics of the included studies are presented in Table 1.

**Table 1:** Characteristics of the included studies. References for all these studies are provided in supplement data 1. RCT: randomized clinical trial.

n.	Study	Year	Type of Study	Country	Number of patients	Age, y	Male, n	Surgery	Fibrinolytic
1.	Abbas S, et al	2020	observational	Pakistan	84	29	41	0	84
2.	Agrawal D, et al	1997	observational	India	42	NR	NR	0	42
3.	Ali A, et al	2021	observational	India	110	39.8	50	0	110
4.	Ahmed F, et al	2022	observational	Pakistan	40	34.5	16	0	40
5.	Ahn H, et al	2008	observational	South Korea	20	42	6	20	0
6.	Brik A, et al	2012	observational	Egypt	92	42	39	92	0
7.	Ali A, et al	2019	observational	Egypt	380	32	112	380	0
8.	Aoyagi S, et al	1996	observational	Japan	20	43.1	5	10	10
9.	Azpitarate J, et al	2001	observational	Spain	33	55	15	14	19
10.	Balram A, et al	1984	observational	India	12	32.5	9	12	0
11.	Bhatia S, et al	2021	observational	India	133	38.4	45	0	133
12.	Bollag L, et al	2001	observational	Switzerland	13	55	6	13	0
13.	Boukarroucha R, et al	2017	observational	Algeria	135	40	NR	135	0
14.	Buttard P, et al	1997	observational	France	21	57	8	21	0
15.	Cáceres FM, et al	2006	observational	Hana	68	40.4	15	0	68
16.	Çakal B, et al	2020	observational	Turkey	85	48.7	32	0	85
17.	Copans H, et al	1980	observational	South Africa	11	NR	4	11	0
18.	Karthikeyan G, et al	2025	RCT	India	79	36.1	39	39	40
19.	Deviri E, et al	1991	observational	South Africa	100	32	32	100	0
20.	Durrleman N, et al	2004	observational	Canada	32	58	7	32	0
21.	Ermis N, et al	2011	observational	Turkey	33	53	13	18	15
22.	Farzaneh K, et al	2020	observational	Iran	65	51.6	26	0	65
23.	Guleria VS, et al	2023	observational	India	21	54.3	7	0	21
24.	Gunduz S, et al	2017	observational	Turkey	27	71	8	0	27
25.	Gupta D, et al	2000	observational	India	110	35.4	52	0	110
26.	Hirachan A, et al	2017	observational	Nepal	23	35	11	0	23
27.	Huang F, et al	2020	observational	China	23	51.2	16	0	23
28.	Issac KN, et al	2023	observational	India	30	40	23	0	30
29.	Kamal Y, et al	2021	observational	Egypt	42	NR	NR	18	24
30.	Karabulut MN, et al	2024	observational	Turkey	27	40.7	5	27	0
31.	Karthikeyan G, et al	2009	observational	India	120	31	67	0	120
32.	Karumuri S, et al	2019	observational	India	66	35.2	23	0	66
33.	Kathirvel D, et al	2018	observational	India	47	33	28	0	52
34.	Katircioglu SF, et al	1999	observational	Turkey	60	37.55	30	60	0
35.	KeuleersS, et al	2011	observational	Belgium	31	59	9	18	13
36.	Khan A, et al	2021	observational	Pakistan	45	35.5	13	0	45
37.	Kiran GR, et al	2021	observational	India	84	41.1	32	0	84
38.	Koca V, et al	2000	observational	Turkey	21	47	7	0	21
39.	Kontos GJ, et al	1989	observational	The USA	27	50	8	27	0
40.	Kothari J, et al	2016	observational	India	65	NR	17	65	0
41.	Kumar S, et al	2001	observational	India	41	NR	14	0	48
42.	Kumar S, et al	2023	observational	India	25	38	12	0	25
43.	Lafci B, et al	2006	observational	Turkey	18	35.9	6	18	0
44.	Ledain LD, et al	1986	observational	France	26	55.6	9	0	28
45.	Lengyel M, et al	2001	observational	Hungary	63	53	22	20	43
46.	Lopez HP, et al	2002	observational	Cuba	15	36	4	0	15
47.	Mahindru S, et al	2021	observational	India	66	40.27	39	0	66
48.	Manandhar R, et al	2022	observational	NEPAL	42	34.9	18	0	42
49.	Mansuri Z, et al	2020	observational	India	130	38.37	43	49	81
50.	Manteiga R, et al	1998	observational	France	19	49	4	0	22
51.	Maria alberto P, et al	2019	observational	Brazil	21	48.2	5	21	0
52.	Martinell J, et al	1991	observational	Spain	57	NR	NR	62	0
53.	Mashali MH, et al	2023	observational	Saudi Arabia	10	1.3	5	0	20
54.	Milne O, et al	2020	observational	Australia	21	36.7	8	0	26
55.	Mishra P, et al	2023	observational	India	41	36.7	32	0	41
56.	Montorsi P, et al	2003	observational	ITALY	17	60	4	0	17
57.	Mottahedi B, et al	2020	observational	Iran	32	55	21	32	0
58.	Nagy A, et al	2009	observational	Hungary	55	55.9	17	0	62
59.	NaqviA, et al	2021	observational	India	40	NR	NR	0	40
60.	Narayan P, et al	2016	observational	INDIA	15	NR	NR	0	19
61.	Nasrabadi A, et al	2023	observational	Iran	81	51.6	35	0	81
62.	Nawale JM, et al	2018	observational	India	21	35.2	8	0	21
63.	Nishanth KR, et al	2020	observational	India	30	41.4	10	0	30
64.	Ogutu P, et al	2007	observational	Germany	11	NR	NR	11	0
65.	Özkan M, et al	2013	observational	Turkey	182	43.2	26	0	220
66.	Özkan M, et al	2013	observational	Turkey	24	29	0	0	28
67.	Özkan M, et al	2015	observational	Turkey	114	49	40	0	120
68.	Ozkan M, et al	2016	observational	Turkey	28	52	10	0	28
69.	Özkan M, et al	2022	observational	Turkey	158	49	55	75	83
70.	Ozkokeli M, et al	2005	observational	Turkey	30	40	12	30	0
71.	Patil S, et al	2019	observational	India	105	39.4	37	0	105
72.	Pavie A, et al	1984	observational	France	34	48	17	34	0
73.	Potwar S, et al	2023	observational	India	50	34	22	0	50
74.	Pradhan A, et al	2019	observational	India	16	40	6	0	16
75.	Purushotama TS, et al	2020	observational	India	56	37.1	27	0	56
76.	Raboi A, et al	2010	observational	Yemen	129	34.8	54	129	0
77.	Rajasekhar D, et al	1994	observational	India	12	39	5	0	13
78.	Raman K, et al	2019	observational	India	127	36	95	61	66
79.	Ramos AI, et al	2003	observational	Brazil	17	41	5	0	17
80.	Rao S, et al	2023	observational	India	86	47.2	28	0	86
81.	Rath DP, et al	2019	observational	India	63	NR	NR	63	0
82.	Reddy NK, et al	1994	observational	India	38	32	20	0	44
83.	Renzulli a, et al	2004	observational	Italy	206	63	57	201	26
84.	Reyna G, et al	1997	observational	Spain	19	47.3	NR	19	0
85.	Roudaut R, et al	1992	observational	France	64	NR	NR	0	75
86.	Roudaut R, et al	2009	observational	France	210	59	95	136	127
87.	Sadeghipour P, et al	2022	RCT	Iran	120	36.3	57	0	120
88.	Sadeghipour P, et al	2024	observational	Iran	135	NR	NR	0	135
89.	Sakr SA, et al	2015	observational	Egypt	20	37	10	0	20
90.	Saleh H, et al	2021	observational	Egypt	97	34.2	36	97	0
91.	Santos H, et al	2021	observational	Mexico	38	47.5	NR	28	10
92.	Separham A, et al	2015	observational	Iran	65	49.4	34	49	16
93.	Shapira Y, et al	2000	observational	Israel	12	58.8	5	0	17
94.	Sharif khan H, et al	2020	observational	Pakistan	64	48.4	23	12	52
95.	Sharma V, et al	2022	observational	India	72	30.1	29	0	72
96.	Sharma V, et al	2012	observational	India	10	40.3	8	0	10
97.	Shojaeifard M, et al	2023	observational	Iran	85	58	33	63	22
98.	Showkathali R, et al	2020	observational	India	44	50	25	0	44
99.	Silber H, et al	1993	observational	The USA	10	66.8	2	0	10
100.	Singh AK, et al	2011	observational	India	86	34.7	38	0	94
101.	Singh S, et al	2015	observational	India	44	27.7	28	0	44
102.	Solorio S, et al	1994	observational	Mexico	10	42.5	1	0	10
103.	Tadesse KD, et al	2023	observational	Ethiopia	11	28	2	0	11
104.	Taljaard JJ, et al	2003	observational	South Africa	20	36	2	20	0
105.	Tareen H, et al	2022	observational	Pakistan	58	32.8	22	0	58
106.	Teshima H, et al	2002	observational	Japan	22	54	7	0	27
107.	Toker ME, et al	2006	observational	Turkey	63	40	21	63	0
108.	Tong AT, et al	2004	observational	USA/Turkey	107	54.2	36	0	107
109.	Tsai KT, et al	1993	observational	Taiwan	10	51	1	10	0
110.	Valenci JS, et al	1999	observational	Mexico	108	45	24	114	0
111.	Valencia JS, et al	2014	observational	Mexico	22	53.7	6	0	22
112.	Vasan RS, et al	1992	observational	India	16	40.8	6	0	16
113.	Vitale N, et al	1994	observational	Italy	28	51	14	20	8
114.	Witchitz S, et al	1980	observational	France	12	51	5	0	13
	sum		–	–	6514	–	2337	2549	4195

### 3.2 Primary and secondary outcome measures

**Fig. 2:**
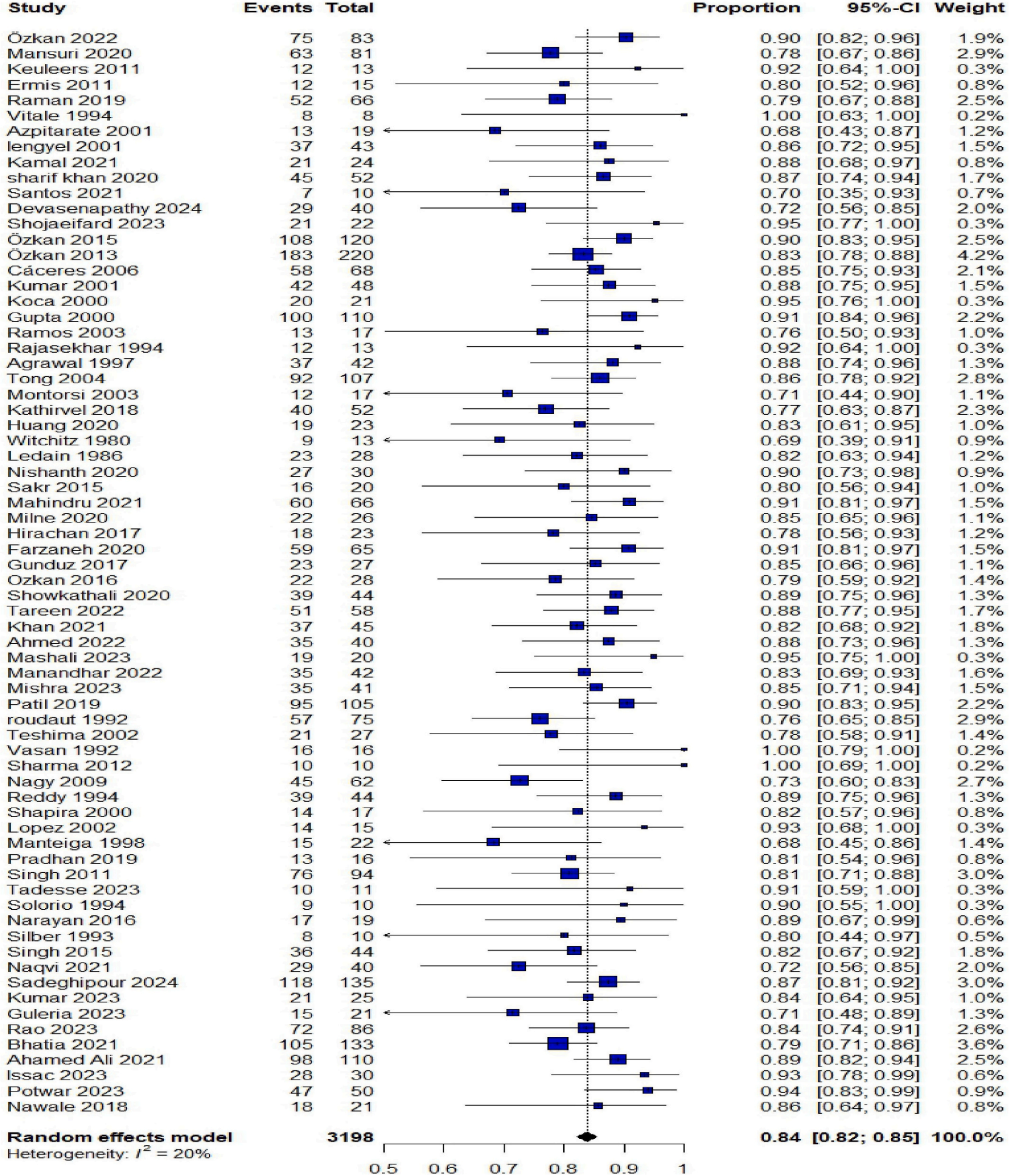
Success rate in fibrinolytic group.

**Fig. 3:**
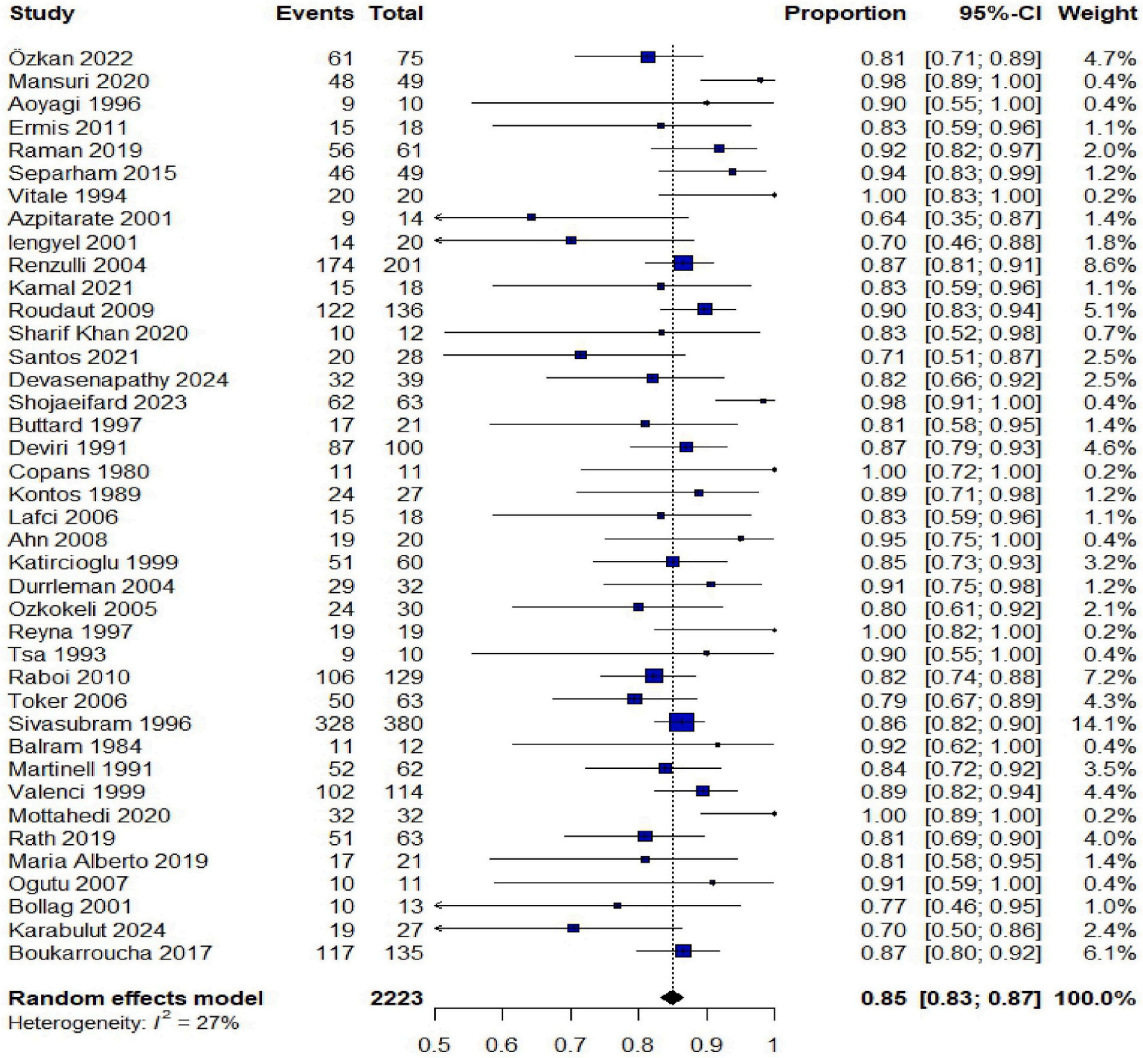
Success rate in surgery group.

**Fig. 4:**
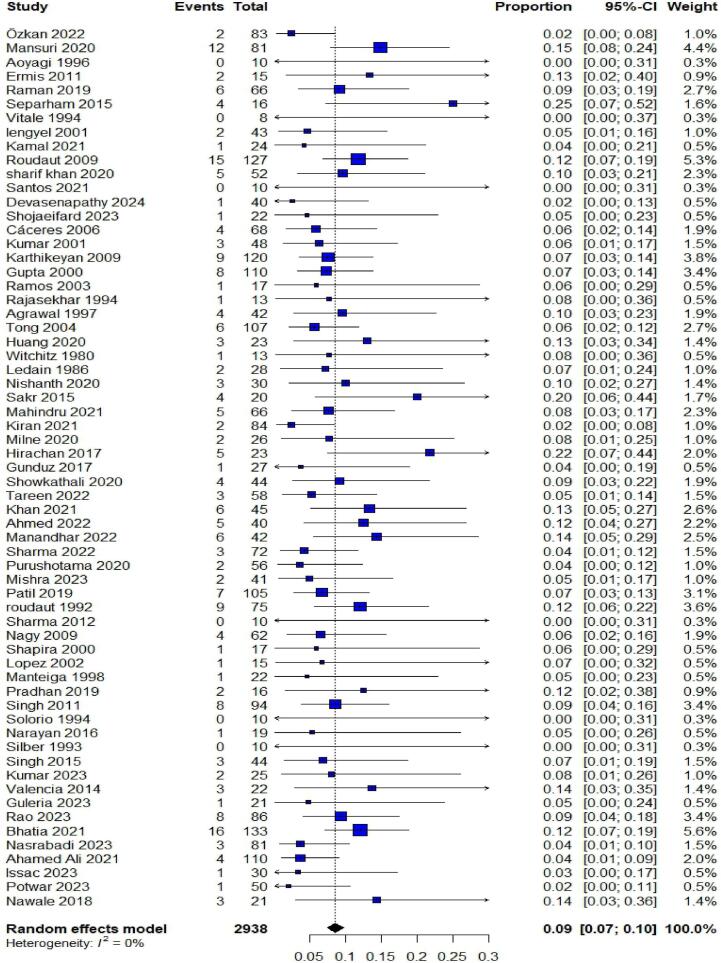
Mortality in fibrinolytic treatment.

**Fig. 5:**
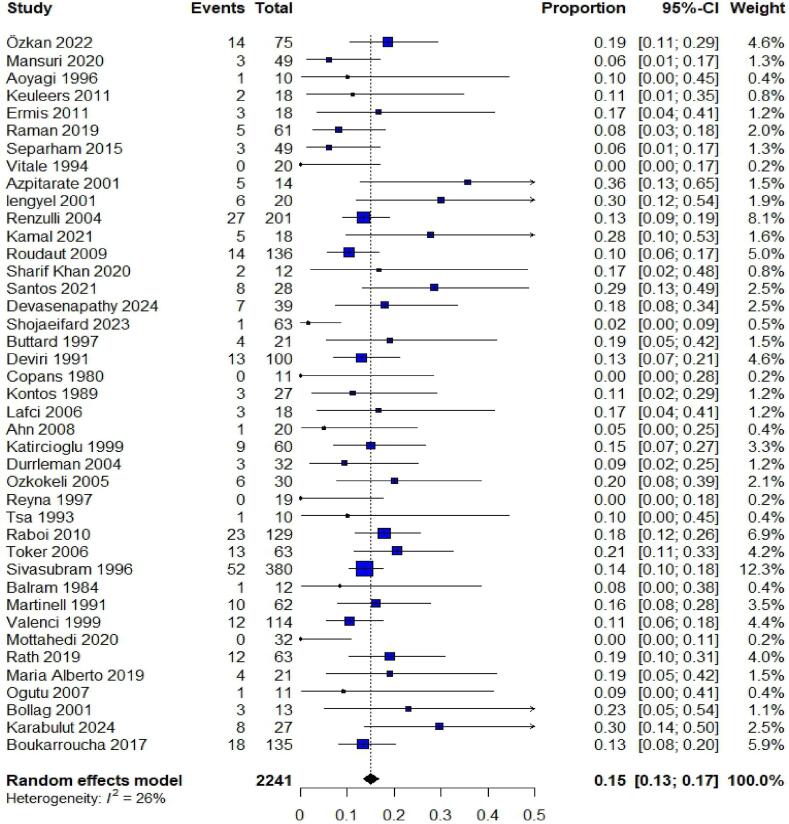
Mortality in surgery treatment.

After analyzing the studies of each group (surgery and thrombolytic), articles with high heterogeneity were excluded. Then, publication bias was examined, and after excluding articles that had the greatest impact on publication bias, it was observed that the success rate in both treatment groups was the same: 83.9% (95% CI: 82.2–85.4; I^2^ = 19.8%) for thrombolysis versus 85.1% (95% CI: 83.3–86.7; I^2^ = 27%) for surgery (*P*-value = 0.607). The mortality rate was lower with thrombolysis (8.5%; 95% CI: 7.5–9.8; I^2^ = 0%) than surgery (15%; 95% CI: 13.4–16.8; I^2^ = 26.3%) (P-value = 0.001). Table 2 summarize outcomes for thrombolytic and surgery groups and compare the two groups respectively. Other secondary outcomes showed no significant differences. Forest plot of success and mortality in fibrinolytic and surgery groups are shown in Fig. 2, Fig. 3, Fig. 4, Fig. 5.

**Table 2:** Success and complications rate of each outcome in each therapy.

Outcome	Treatment	Number of studies⁎	Pooled rate (95%CI)	I^2^	P-value for differences
Success rate	Surgery	40	85.1 (0.833, 0.867)	27	0.607
	Thrombolytic therapy	70	83.9 (0.822, 0.854)	20	
In-hospital mortality	Surgery	41	15 (0.134, 0.168)	26	**0.001**
	Thrombolytic therapy	63	8.5 (0.075, 0.098)	0	
Nonfatal intracranial hemorrhage	Surgery	30	1.4 (0.009, 0.023)	0	0.261
	Thrombolytic therapy	85	2.4 (0.019, 0.030)	0	
Nonfatal major bleeding	Surgery	30	4.3 (0.030, 0.062)	22	0.520
	Thrombolytic therapy	85	3.2 (0.027, 0.039)	0	
Nonfatal stroke and transient ischemic attack	Surgery	30	2.8 (0.020–0.038	0	0.060
	Thrombolytic therapy	85	5.9 (0.045, 0.066)	24.8	
Non-CNS embolic event	Surgery	30	2.5 (0.016–0.040)	24.9	0.661
	Thrombolytic therapy	85	3.8 (0.028, 0.046)	17.3	

⁎ Number of studies after excluding articles with high heterogeneity and articles that had the greatest impact on publication bias. CNS: central nervous system.

In the slow/very slow thrombolytic infusion subgroup analysis (11 studies), after excluding articles with high heterogeneity and articles that had the greatest impact on publication bias, the success rate was 91.4% (95% CI: 87–94; I^2^ = 44.9%) and the mortality was 2.1% (95% CI: 1.2–3.3; I^2^ = 0%) (Table 3).

**Table 3:** Outcomes in slow/very slow infusion group.

Outcome	Chi2			$I^{2}$	$\tau^{2}$	Pooled rate (95%CI)
	P-Value	df	Q			
**Success rate**	0.091	6	10.9	44.9%	0.142	0.914 (0.873–0.943)
**In-hospital mortality**	0.876	10	5.2	0%	0	0.021 (0.012–0.033)
**Nonfatal intracranial hemorrhage**	0.998	10	4.4	0%	0	0.017 (0.010–0.029)
**Nonfatal major bleeding**	0.965	10	3.5	0%	0	0.020 (0.012–0.033)
**Nonfatal stroke and transient ischemic attack**	0.412	10	10.3	3.2%	0.026	0.029 (0.018–0.046)
**Non-CNS embolic event**	0.863	10	5.3	0%	0	0.019 (0.011–0.032)

CNS: central nervous system.

## 4 Discussion

In this systematic review and meta-analysis comparing fibrinolytic therapy with surgery for prosthetic valve thrombosis, the overall success rate (defined as restoration of normal valve function without additional intervention) showed no statistically significant difference between the two approaches. Although the overall success rates were comparable, these findings are primarily derived from observational studies with inherent limitations. Therefore, the results should be interpreted with caution, and high-quality randomized controlled trials are still needed to confirm the optimal treatment strategy.

This equivalence in efficacy is consistent with prior high-quality meta-analyses. Karthikeyan et al. [6] reported success rates of 69.7% for fibrinolysis and 86.5% for surgery (*p* = 0.06), while Castilho et al. [7] found 80.7% vs. 81.9% (*p* = 0.72). More recently, Chopard et al. [8] (the largest contemporary analysis including over 1389 patients) confirmed no significant difference (77.9% vs. 80.4%, *p* = 0.1). Our findings thus reinforce a decade-long consensus: both modalities achieve comparable thrombus resolution. This aligns with prior evidence, including registry data from the European society of cardiology/European association for cardio-thoracic surgery (ESC/EACTS), which position surgery as the historical gold standard while acknowledging fibrinolysis as a viable alternative in high-surgical-risk patients [3].

However, a clinically meaningful divergence emerged in in-hospital mortality, which was significantly lower with fibrinolysis. This survival advantage is partially supported by prior meta-analyses, with heterogeneous statistical significance: Castilho et al. [7] reported 6.6% vs. 18.1% (*p* < 0.001), Chopard et al. [8] reported 10.8% vs. 15.3% (p < 0.001), and Karthikeyan et al. [6] reported 9% vs. 13.5% (*p* = 0.24), a non-significant trend likely due to smaller sample size (*n* = 690) and era-specific surgical outcomes. Despite the non-significance in Karthikeyan et al., the directional consistency across all three reviews and our updated pooled estimate strengthens the hypothesis that fibrinolysis reduces early mortality by avoiding open-chest surgical complications, including coagulopathy, massive bleeding, mediastinitis, or multi-organ failure secondary to cardiopulmonary bypass. In our study, while success rates were statistically similar between the two strategies, in-hospital mortality was significantly lower with fibrinolytic therapy. Given that mortality is the most clinically relevant hard endpoint, this finding carries substantial weight in decision-making, particularly for patients with high surgical risk.

No significant differences were observed in other complications in our study, including nonfatal stroke and transient ischemic attack (TIA), non-central nervous system (CNS) embolism, nonfatal intracranial hemorrhage (ICH) and nonfatal major bleeding, reinforcing the short-term safety profile of fibrinolysis, particularly in centres with limited emergent surgical capacity.

### 4.1 Descriptive subgroup analysis

Patients receiving slow or very-slow infusion fibrinolysis demonstrated higher crude success (91.4%) and lower in-hospital mortality (2.1%) than both the overall fibrinolysis cohort (83.9% success, 8.5% mortality) and the surgical arm (85.1% success, 15% mortality). While no *p*-values were calculated due to lack of direct head-to-head comparison in primary studies, this pattern suggests a dose-duration optimization effect. Several mechanistic and pharmacokinetic principles likely underlie this advantage:

1. Minimized systemic plasmin activation and reduces systemic fibrinogen depletion: rapid bolus administration generates high peak plasma concentrations, triggering widespread plasmin generation and increasing risks of major bleeding (including intracranial) and distal embolization. In contrast, slow infusion maintains steady-state drug levels, favoring localized fibrinolysis at the valve thrombus while preserving systemic hemostasis [9], [10].
2. Enhanced penetration into organized thrombus: Mechanical valve thrombi are often mature and fibrotic, resistant to bolus therapy. Prolonged infusion allows gradual drug penetration into deeper thrombus layers, analogous to the established efficacy of slow thrombolysis in venous thromboembolism [9], [11].
3. Supporting clinical evidence: These results corroborate major studies such as PROMETEE [12] and HATTUSHA [13] support its clinical efficacy and safety of slow/very slow infusion of fibrinolytics, which reported >90% success and < 5% mortality using slow-infusion protocols in PVT. Outcomes with these low-dose slow or very slow regimens are more favorable than those observed in earlier (before 2013) fibrinolytic studies with rapid infusion rates and higher doses. It seems, as demonstrated in high-quality prospective studies such as HATTUSHA [13], standardized low-dose slow/very slow infusion of fibrinolytics appears to yield higher success rates and improved safety compared with older regimens included in our pooled analysis.

The present results confirm the American Heart Association/American College of Cardiology (AHA/ACC) recommendations by providing additional evidence in favor of systemic thrombolysis over surgery for the management of PVT. The American guidelines recommend the use of slow-infusion, low-dose fibrinolytic therapy and reserve surgery for patients with New York Heart Association (NYHA) functional class IV, recurrent valve thrombosis, large thrombi (>0.8 cm2), and contraindications to thrombolysis [2]. In contrast, the current ESC guidelines generally favor urgent/emergency valve surgery as the first-line treatment for obstructive left-sided PVT, while reserving thrombolysis for high surgical risk cases, unavailable surgery, or specific scenarios [3]. These differences likely stem from varying interpretations of observational data, bleeding risks with older thrombolytic regimens, and surgical expertise availability. Recent meta-analyses and large registries have provided additional support for thrombolysis as a viable and potentially safer option in non-critically ill patients. Dedicated RCTs comparing slow/very slow infusion fibrinolysis versus surgery are warranted to confirm whether the observed high success rate translates to superior risk-adjusted outcomes. The SAFE-PVT [14] is the first and only RCT to compare urgent surgery versus fibrinolytic for the treatment of PVT. The results of our study are supported by the results of the SAFE-PVT trial. The result of the SAFE-PVT study showed complete clinical response at discharge was 71.8% in surgery group and 67.5% in fibrinolytic group (OR = 1.22,95% CI 0.47–3.19, *P* = 0.689) and the primary composite safety outcome (composite of death, non-fatal stroke, non-fatal major bleeding and non-CNS systemic embolism) was 28.2% in surgery group and 7.5% in fibrinolytic group (OR = 5.14, 95% CI 1.28–20.59, *P* = 0.021).

### 4.2 Study limitations

We acknowledge several important limitations inherent to the underlying evidence. Most included studies were retrospective observational series, which introduces significant confounding by indication and selection bias. To overcome this limitation, we performed sensitivity analyses to find the source of between-study heterogeneity for study outcomes.

Patients in the surgical group had a higher proportion of NYHA class IV (38% vs 25%), and important covariates such as thrombus size, pannus versus thrombus differentiation, hemodynamic instability, and time since valve implantation were inconsistently reported. Most studies also reported only in-hospital outcomes with variable definitions of complications, limiting long-term interpretation.

Furthermore, fibrinolytic regimens varied widely, including older rapid high-dose protocols, which likely underestimate the performance of modern low-dose slow/very slow infusion regimens, as elegantly demonstrated in the HATTUSHA study [13]. These factors may bias pooled thrombolytic estimates toward less favorable outcomes compared with contemporary prospective cohorts that are well-designed and use modern standard protocols.

Although, the superior crude outcomes in the slow/very slow subgroup must be interpreted cautiously, the consistency of mortality reduction with fibrinolysis across four meta-analyses [6–8 and present study] strengthens its role as first-line therapy in non-critical PVT, particularly when surgical risk is elevated.

Additionally, important surgical risk scores (e.g., EuroSCORE) were insufficiently reported in the primary studies, preventing subgroup analyses based on these factors. This remains an important area for future research.

## 5 Conclusions

This systematic review suggests that fibrinolytics are a reasonable option alongside surgery for left-sided PVT, with similar success rates and lower mortality. However, due to observational evidence, results should be interpreted cautiously and RCTs are needed.

## CRediT authorship contribution statement

**Babak Bagheri:** Writing – review & editing, Supervision, Conceptualization. **Sahar Eskandari:** Writing – review & editing, Data curation. **Saeid Soltani:** Writing – original draft, Investigation, Conceptualization. **Rozita Jalalian:** Investigation, Data curation, Conceptualization. **Jamshid Yazdani Charati:** Software, Project administration, Methodology. **Masoud Moradi:** Software, Project administration, Methodology. **Mohammadreza Iranian:** Writing – original draft, Supervision, Investigation, Conceptualization.

## Responsibility statement

The authors take responsibility for all aspects of the reliability and freedom from bias of the data presented and their discussed interpretation.

## Ethical statement

This systematic review was conducted based on publicly available literature and does not involve human participants, animal subjects, or primary data collection. As no primary data was collected, and all included studies adhered to ethical standards outlined by their respective institutional review boards, this review does not require new ethical approval. The authors declare no conflicts of interest, and the study adheres to the ethical guidelines for publishing in peer-reviewed journals. All references were properly cited, and data were synthesized transparently and accurately without any manipulation. Also, details about analysis are reserved by the authors and will be provided by the corresponding author upon reasonable request.

## Declaration of Generative AI and AI-assisted technologies in the writing process

In the preparation of the discussion section, the artificial intelligence tool Grok (developed by xAI) was used to generate initial drafting suggestions and assist with idea formulation. The final content was extensively reviewed, revised, and approved by the authors, who assume full responsibility for the accuracy, integrity, and originality of the work. The AI tool was not involved in study design, data acquisition, analysis of results or interpretation.

## Funding statement

The authors declare that no funds, grants, or other support were received during the preparation of this manuscript.

## Declaration of competing interest

All of the authors mention that there is no conflict of interest in the study.
