# Supplementary material for: Thrombolytic therapy versus cardiac surgery for left-sided prosthetic heart valve thrombosis: A systematic review and meta-analysis
Source: Am Heart J Plus. 2026 Jul 29;69:100849. doi: 10.1016/j.ahjo.2026.100849 (PMC13451796; doi:10.1016/j.ahjo.2026.100849)
Supplement: Supplementary file 1 [file mmc1.docx]

| **Table S1: Search strategy** |  |
| --- | --- |
| **Query: pubmed** | **Results (No.)** |
| (((((prosthetic heart valve) OR (mechanical heart valve)) AND (thrombosis)) OR (((prosthetic heart valve thrombosis) OR (prosthetic valve thrombosis)) OR (mechanical heart valve thrombosis))) AND ((((surgery) OR (valve replacement)) OR (thrombectomy)) OR (heart valve replacement))) OR ((((((((((((((Thrombolytic Therapy) OR (Fibrinolytic Agents)) OR (Streptokinase)) OR (Anistreplase)) OR (Tissue Plasminogen Activator)) OR (Tenecteplase)) OR (Urokinase-Type Plasminogen Activator)) OR (urokinase)) OR (t‐ PA)) OR (Alteplase)) OR (Fibrinolytic therapy)) OR (fibrinolysis)) OR (reteplase)) AND ((((prosthetic heart valve) OR (mechanical heart valve)) AND (thrombosis)) OR (((prosthetic heart valve thrombosis) OR (prosthetic valve thrombosis)) OR (mechanical heart valve thrombosis)))) | 4614 |
| **Query: Scopus** | Results (No.) |
| ( ( TITLE-ABS-KEY ( thrombolytic AND therapy ) OR TITLE-ABS-KEY ( fibrinolytic AND agents ) OR TITLE-ABS-KEY ( streptokinase ) OR TITLE-ABS-KEY ( anistreplase ) OR TITLE-ABS-KEY ( tissue AND plasminogen AND activator ) OR TITLE-ABS-KEY ( tenecteplase ) OR TITLE-ABS-KEY ( urokinase-type AND plasminogen AND activator ) OR TITLE-ABS-KEY ( urokinase ) OR TITLE-ABS-KEY ( t&#8208; AND pa ) OR TITLE-ABS-KEY ( alteplase ) OR TITLE-ABS-KEY ( fibrinolytic AND therapy ) OR TITLE-ABS-KEY ( fibrinolysis ) OR TITLE-ABS-KEY ( reteplase ) ) ) AND ( ( ( TITLE-ABS-KEY ( prosthetic AND heart AND valve AND thrombosis ) OR TITLE-ABS-KEY ( prosthetic AND valve AND thrombosis ) OR TITLE-ABS-KEY ( mechanical AND heart AND valve AND thrombosis ) OR TITLE-ABS-KEY ( mechanical AND valve AND thrombosis ) ) ) OR ( ( TITLE-ABS-KEY ( prosthetic AND heart AND valve AND thrombosis ) OR TITLE-ABS-KEY ( prosthetic AND valve AND thrombosis ) OR TITLE-ABS-KEY ( mechanical AND heart AND valve AND thrombosis ) OR TITLE-ABS-KEY ( mechanical AND valve AND thrombosis ) ) ) ) AND ( LIMIT-TO ( DOCTYPE , "ar" ) ) | 2825 |
| **Query: Scopus** | **Results (No.)** |
| ( ( TITLE-ABS-KEY ( surgery ) OR TITLE-ABS-KEY ( valve AND replacement ) OR TITLE-ABS-KEY ( thrombectomy ) ) ) AND ( ( TITLE-ABS-KEY ( prosthetic AND heart AND valve AND thrombosis ) OR TITLE-ABS-KEY ( prosthetic AND valve AND thrombosis ) OR TITLE-ABS-KEY ( mechanical AND heart AND valve AND thrombosis ) OR TITLE-ABS-KEY ( mechanical AND valve AND thrombosis ) ) ) AND ( LIMIT-TO ( DOCTYPE , "ar" ) ) | 707 |
| **Query:Embase** | **Results (No.)** |
| (('prosthetic heart valve':ab,ti OR 'mechanical heart valve':ab,ti) AND 'thrombosis':ab,ti OR 'prosthetic heart valve thrombosis':ab,ti OR 'prosthetic valve thrombosis':ab,ti OR 'mechanical heart valve thrombosis':ab,ti) AND ('surgery':ab,ti OR 'valve replacement':ab,ti OR 'thrombectomy':ab,ti OR 'heart valve replacement':ab,ti) OR (('thrombolytic therapy':ab,ti OR 'fibrinolytic agents':ab,ti OR 'streptokinase':ab,ti OR 'anistreplase':ab,ti OR 'tissue plasminogen activator':ab,ti OR 'tenecteplase':ab,ti OR 'urokinase-type plasminogen activator':ab,ti OR 'urokinase':ab,ti OR 't‐ pa':ab,ti OR 'alteplase':ab,ti OR 'fibrinolytic therapy':ab,ti OR 'fibrinolysis':ab,ti OR 'reteplase':ab,ti) AND (('prosthetic heart valve':ab,ti OR 'mechanical heart valve':ab,ti) AND 'thrombosis':ab,ti OR 'prosthetic heart valve thrombosis':ab,ti OR 'prosthetic valve thrombosis':ab,ti OR 'mechanical heart valve thrombosis':ab,ti)) | 805 |

| Table S2: Risk of bias assessment of included studies according to National Heart, Lung, and Blood Institute | | | | | | | | | | | | | | | |
| --- | --- | --- | --- | --- | --- | --- | --- | --- | --- | --- | --- | --- | --- | --- | --- |
| study | | Item | | | | | | | | | | | | | |
|  |  | 1 | 2 | 3 | 4 | 5 | 6 | 7 | 8 | 9 | 10 | 11 | 12 | 13 | 14 |
| 1. | Abbas2020 | Yes | Yes | Yes | Yes | No | Yes | Yes | No | Yes | NA | Yes | NA | Yes | No |
| 2. | Agrawal1997 | Yes | Yes | Yes | Yes | No | Yes | Yes | No | Yes | NA | Yes | NA | Yes | No |
| 3. | Ahamed Ali2021 | Yes | Yes | Yes | Yes | No | Yes | Yes | No | Yes | NA | Yes | NA | Yes | No |
| 4. | Ahmed2022 | Yes | Yes | Yes | Yes | No | Yes | Yes | No | Yes | NA | Yes | NA | Yes | No |
| 5. | Ahn2008 | Yes | Yes | Yes | Yes | No | Yes | Yes | No | Yes | NA | Yes | NA | Yes | No |
| 6. | Alaa2012 | Yes | Yes | Yes | Yes | No | Yes | Yes | NA | Yes | NA | Yes | NA | Yes | No |
| 7. | Ali2019 | Yes | Yes | Yes | Yes | No | Yes | Yes | NA | Yes | NA | Yes | NA | Yes | No |
| 8. | Aoyaji1996 | Yes | Yes | Yes | Yes | No | Yes | Yes | No | Yes | NA | Yes | NA | Yes | Yes |
| 9. | Azpitarate2001 | Yes | Yes | Yes | Yes | No | Yes | Yes | No | Yes | NA | Yes | NA | Yes | No |
| 10. | Balram1984 | Yes | Yes | Yes | Yes | No | Yes | Yes | NA | Yes | NA | Yes | NA | Yes | No |
| 11. | Bhatia2021 | Yes | Yes | Yes | Yes | No | Yes | Yes | No | Yes | NA | Yes | NA | Yes | Yes |
| 12. | Bollag2001 | Yes | Yes | Yes | Yes | No | Yes | Yes | NA | Yes | NA | Yes | NA | Yes | No |
| 13. | Boukarroucha2017 | Yes | Yes | Yes | Yes | No | Yes | Yes | NA | Yes | NA | Yes | NA | Yes | No |
| 14. | Buttard1997 | Yes | Yes | Yes | Yes | No | Yes | Yes | No | Yes | NA | Yes | NA | Yes | No |
| 15. | Cáceres2006 | Yes | Yes | Yes | Yes | No | Yes | Yes | No | Yes | NA | Yes | NA | Yes | No |
| 16. | Çakal2020 | Yes | Yes | Yes | Yes | No | Yes | Yes | No | Yes | NA | Yes | NA | Yes | No |
| 17. | Copans1980 | Yes | Yes | Yes | Yes | No | Yes | Yes | No | Yes | NA | Yes | NA | Yes | No |
| 18. | Devasenapathy2024 | Yes | Yes | Yes | No | No | Yes | Yes | Yes | Yes | Yes | Yes | Yes | Yes | Yes |
| 19. | DEVIRI1991 | Yes | Yes | Yes | Yes | No | Yes | Yes | No | Yes | NA | Yes | NA | Yes | Yes |
| 20. | Durrleman2004 | Yes | Yes | Yes | Yes | No | Yes | Yes | NA | Yes | NA | Yes | NA | Yes | No |
| 21. | Ermis2011 | Yes | Yes | Yes | Yes | No | Yes | Yes | No | Yes | NA | Yes | NA | Yes | No |
| 22. | Farzaneh2020 | Yes | Yes | Yes | Yes | No | Yes | Yes | No | Yes | NA | Yes | NA | Yes | No |
| 23. | Guleria2023 | Yes | Yes | Yes | Yes | No | Yes | Yes | No | Yes | NA | Yes | NA | Yes | Yes |
| 24. | Gunduz2017 | Yes | Yes | Yes | Yes | No | Yes | Yes | No | Yes | NA | Yes | NA | Yes | Yes |
| 25. | Gupta2000 | Yes | Yes | Yes | Yes | No | Yes | Yes | No | Yes | NA | Yes | NA | Yes | Yes |
| 26. | Hirachan2017 | Yes | Yes | Yes | Yes | No | Yes | Yes | No | Yes | NA | Yes | NA | Yes | No |
| 27. | Huang2020 | Yes | Yes | Yes | Yes | No | Yes | Yes | No | Yes | NA | Yes | NA | Yes | No |
| 28. | Issac2023 | Yes | Yes | Yes | Yes | No | Yes | Yes | No | Yes | NA | Yes | NA | Yes | No |
| 29. | Kamal2021 | Yes | Yes | Yes | Yes | No | Yes | Yes | No | Yes | NA | Yes | NA | Yes | No |
| 30. | Karabulut2024 | Yes | Yes | Yes | Yes | No | Yes | Yes | NA | Yes | NA | Yes | NA | Yes | No |
| 31. | Karthikeyan2009 | Yes | Yes | Yes | Yes | No | Yes | Yes | Yes | Yes | NA | Yes | NA | Yes | Yes |
| 32. | Karumuri2019 | Yes | Yes | Yes | Yes | No | Yes | Yes | No | Yes | NA | Yes | NA | Yes | No |
| 33. | Kathirvel2018 | Yes | Yes | Yes | Yes | No | Yes | Yes | Yed | Yes | NA | Yes | NA | Yes | No |
| 34. | Katircioglu1999 | Yes | Yes | Yes | Yes | No | Yes | Yes | No | Yes | NA | Yes | NA | Yes | No |
| 35. | Keuleers2011 | Yes | Yes | Yes | Yes | No | Yes | Yes | No | Yes | NA | Yes | NA | Yes | No |
| 36. | Khan2021 | Yes | Yes | Yes | Yes | No | Yes | Yes | No | Yes | NA | Yes | NA | Yes | No |
| 37. | Kiran2021 | Yes | Yes | Yes | Yes | No | Yes | Yes | Yes | Yes | NA | Yes | NA | Yes | No |
| 38. | Koca2000 | Yes | Yes | Yes | Yes | No | Yes | Yes | No | Yes | NA | Yes | NA | Yes | No |
| 39. | Kontos1989 | Yes | Yes | Yes | Yes | No | Yes | Yes | No | Yes | NA | Yes | NA | Yes | No |
| 40. | Kothari2016 | Yes | Yes | Yes | Yes | No | Yes | Yes | NA | Yes | NA | Yes | NA | Yes | No |
| 41. | Kumar2001 | Yes | Yes | Yes | Yes | No | Yes | Yes | No | Yes | NA | Yes | NA | Yes | No |
| 42. | Kumar2023 | Yes | Yes | Yes | Yes | No | Yes | Yes | No | Yes | NA | Yes | NA | Yes | No |
| 43. | Lafci2006 | Yes | Yes | Yes | Yes | No | Yes | Yes | No | Yes | NA | Yes | NA | Yes | No |
| 44. | Ledian1986 | Yes | Yes | Yes | Yes | No | Yes | Yes | No | Yes | NA | Yes | NA | Yes | No |
| 45. | Lengyel2001 | Yes | No | Yes | Yes | No | Yes | Yes | No | Yes | NA | Yes | NA | Yes | No |
| 46. | Lopez2002 | Yes | Yes | Yes | Yes | No | Yes | Yes | No | Yes | NA | Yes | NA | Yes | No |
| 47. | Mahindru2021 | Yes | Yes | Yes | Yes | No | Yes | Yes | No | Yes | NA | Yes | NA | Yes | No |
| 48. | Manandhar2022 | Yes | Yes | Yes | Yes | No | Yes | Yes | No | Yes | NA | Yes | NA | Yes | Yes |
| 49. | Mansuri2020 | Yes | Yes | Yes | Yes | No | Yes | Yes | No | Yes | NA | Yes | NA | Yes | Yes |
| 50. | Manteiga1998 | Yes | Yes | Yes | Yes | No | Yes | Yes | No | Yes | NA | Yes | NA | Yes | No |
| 51. | Maria alberto2019 | Yes | Yes | Yes | Yes | No | Yes | Yes | NA | Yes | NA | Yes | NA | Yes | No |
| 52. | Martinell1991 | Yes | Yes | Yes | Yes | No | Yes | Yes | NA | Yes | NA | Yes | NA | Yes | No |
| 53. | Mashali2023 | Yes | Yes | Yes | Yes | No | Yes | Yes | No | Yes | NA | Yes | NA | Yes | No |
| 54. | Milne2020 | Yes | Yes | Yes | Yes | No | Yes | Yes | No | Yes | NA | Yes | NA | Yes | No |
| 55. | Mishra2023 | Yes | Yes | Yes | Yes | No | Yes | Yes | Yes | Yes | NA | Yes | NA | Yes | Yes |
| 56. | Montorsi2003 | Yes | Yes | Yes | Yes | No | Yes | Yes | No | Yes | NA | Yes | NA | Yes | No |
| 57. | Mottahedi2020 | Yes | Yes | Yes | Yes | No | Yes | Yes | NA | Yes | NA | Yes | NA | Yes | No |
| 58. | Nagy2009 | Yes | Yes | Yes | Yes | No | Yes | Yes | No | Yes | NA | Yes | NA | Yes | Yes |
| 59. | Naqvi2021 | Yes | Yes | Yes | Yes | No | Yes | Yes | No | Yes | NA | Yes | NA | Yes | No |
| 60. | Narayan2016 | Yes | Yes | Yes | Yes | No | Yes | Yes | No | Yes | NA | Yes | NA | Yes | No |
| 61. | Nasrabadi2023 | Yes | Yes | Yes | Yes | No | Yes | Yes | No | Yes | NA | Yes | NA | Yes | No |
| 62. | Nawale2018 | Yes | Yes | Yes | Yes | No | Yes | Yes | No | Yes | NA | Yes | NA | Yes | No |
| 63. | Nishanth2020 | Yes | Yes | Yes | Yes | No | Yes | Yes | No | Yes | NA | Yes | NA | Yes | No |
| 64. | Ogutu2007 | Yes | Yes | Yes | Yes | No | Yes | Yes | NA | Yes | NA | Yes | NA | Yes | No |
| 65. | Özkan2013 | Yes | Yes | Yes | Yes | No | Yes | Yes | Yes | Yes | NA | Yes | NA | Yes | Yes |
| 66. | Özkan2013 | Yes | Yes | Yes | Yes | No | Yes | Yes | Yes | Yes | NA | Yes | NA | Yes | No |
| 67. | Özkan2015 | Yes | Yes | Yes | Yes | No | Yes | Yes | Yes | Yes | NA | Yes | NA | Yes | Yes |
| 68. | Ozkan2016 | Yes | Yes | Yes | Yes | No | Yes | Yes | No | Yes | NA | Yes | NA | Yes | Yes |
| 69. | Özkan2022 | Yes | Yes | Yes | Yes | No | Yes | Yes | Yes | Yes | NA | Yes | NA | Yes | No |
| 70. | Ozkokeli2005 | Yes | Yes | Yes | Yes | No | Yes | Yes | NA | Yes | NA | Yes | NA | Yes | No |
| 71. | Patil2019 | Yes | Yes | Yes | Yes | No | Yes | Yes | No | Yes | NA | Yes | NA | Yes | No |
| 72. | PAVIE1984 | Yes | Yes | Yes | Yes | No | Yes | Yes | NA | Yes | NA | Yes | NA | Yes | No |
| 73. | Potwar2023 | Yes | Yes | Yes | Yes | No | Yes | Yes | No | Yes | NA | Yes | NA | Yes | No |
| 74. | Pradhan2019 | Yes | Yes | Yes | Yes | No | Yes | Yes | No | Yes | NA | Yes | NA | Yes | No |
| 75. | Purushotama 2020 | Yes | Yes | Yes | Yes | No | Yes | Yes | No | Yes | NA | Yes | NA | Yes | No |
| 76. | Raboi2010 | Yes | Yes | Yes | Yes | No | Yes | Yes | NA | Yes | NA | Yes | NA | Yes | No |
| 77. | Rajasekhar1994 | Yes | Yes | Yes | Yes | No | Yes | Yes | No | Yes | NA | Yes | NA | Yes | No |
| 78. | Raman2019 | Yes | Yes | Yes | Yes | No | Yes | Yes | No | Yes | NA | Yes | NA | Yes | No |
| 79. | Ramos2003 | Yes | Yes | Yes | Yes | No | Yes | Yes | No | Yes | NA | Yes | NA | Yes | No |
| 80. | Rao2023 | Yes | Yes | Yes | Yes | No | Yes | Yes | No | Yes | NA | Yes | NA | Yes | No |
| 81. | Rath2019 | Yes | Yes | Yes | Yes | No | Yes | Yes | NA | Yes | NA | Yes | NA | Yes | No |
| 82. | Reddy1994 | Yes | Yes | Yes | Yes | No | Yes | Yes | No | Yes | NA | Yes | NA | Yes | No |
| 83. | Renzulli2004 | Yes | Yes | Yes | Yes | No | Yes | Yes | No | Yes | NA | Yes | NA | Yes | No |
| 84. | Reyna1997 | Yes | Yes | Yes | Yes | No | Yes | Yes | NA | Yes | NA | Yes | NA | Yes | No |
| 85. | Roudaut1992 | Yes | Yes | Yes | Yes | No | Yes | Yes | No | Yes | NA | Yes | NA | Yes | No |
| 86. | Roudaut2009 | Yes | Yes | Yes | Yes | No | Yes | Yes | No | Yes | NA | Yes | NA | Yes | No |
| 87. | Sadeghipour2022 | Yes | Yes | Yes | No | No | Yes | Yes | Yes | Yes | Yes | Yes | No | Yes | Yes |
| 88. | Sadeghipour2024 | Yes | Yes | Yes | Yes | No | Yes | Yes | Yes | Yes | NA | Yes | NA | Yes | Yes |
| 89. | Sakr2015 | Yes | Yes | Yes | Yes | No | Yes | Yes | No | Yes | NA | Yes | NA | Yes | Yes |
| 90. | Saleh2021 | Yes | Yes | Yes | Yes | No | Yes | Yes | NA | Yes | NA | Yes | NA | Yes | No |
| 91. | Santos2021 | Yes | Yes | Yes | Yes | No | Yes | Yes | No | Yes | NA | Yes | NA | Yes | No |
| 92. | Separham2015 | Yes | Yes | Yes | Yes | No | Yes | Yes | No | Yes | NA | Yes | NA | Yes | No |
| 93. | Shapira2000 | Yes | Yes | Yes | Yes | No | Yes | Yes | No | Yes | NA | Yes | NA | Yes | No |
| 94. | Sharif khan2020 | Yes | Yes | Yes | Yes | No | Yes | Yes | No | Yes | NA | Yes | NA | Yes | No |
| 95. | Sharma 2022 | Yes | Yes | Yes | Yes | No | Yes | Yes | No | Yes | NA | Yes | NA | Yes | No |
| 96. | Sharma2012 | Yes | Yes | Yes | Yes | No | Yes | Yes | No | Yes | NA | Yes | NA | Yes | No |
| 97. | Shojaeifard2023 | Yes | Yes | Yes | Yes | No | Yes | Yes | No | Yes | NA | Yes | NA | Yes | Yes |
| 98. | Showkathali2020 | Yes | Yes | Yes | Yes | No | Yes | Yes | No | Yes | NA | Yes | NA | Yes | No |
| 99. | Silber1993 | Yes | Yes | Yes | Yes | No | Yes | Yes | No | Yes | NA | Yes | NA | Yes | No |
| 100. | Singh2011 | Yes | Yes | Yes | Yes | No | Yes | Yes | No | Yes | NA | Yes | NA | Yes | Yes |
| 101. | Singh2015 | Yes | Yes | Yes | Yes | No | Yes | Yes | No | Yes | NA | Yes | NA | Yes | No |
| 102. | Solorio 1994 | Yes | Yes | Yes | Yes | No | Yes | Yes | No | Yes | NA | Yes | NA | Yes | No |
| 103. | Tadesse2023 | Yes | Yes | Yes | Yes | No | Yes | Yes | No | Yes | NA | Yes | NA | Yes | No |
| 104. | TALJAARD2003 | Yes | Yes | Yes | Yes | No | Yes | Yes | NA | Yes | NA | Yes | NA | Yes | No |
| 105. | TAREEN2022 | Yes | Yes | Yes | Yes | No | Yes | Yes | No | Yes | NA | Yes | NA | Yes | No |
| 106. | Teshima2002 | Yes | Yes | Yes | Yes | No | Yes | Yes | No | Yes | NA | Yes | NA | Yes | No |
| 107. | Toker2006 | Yes | Yes | Yes | Yes | No | Yes | Yes | NA | Yes | NA | Yes | NA | Yes | No |
| 108. | Tong2004 | Yes | Yes | Yes | Yes | No | Yes | Yes | No | Yes | NA | Yes | NA | Yes | Yes |
| 109. | Tsa1993 | Yes | Yes | Yes | Yes | No | Yes | Yes | NA | Yes | NA | Yes | NA | Yes | No |
| 110. | Valenci1999 | Yes | Yes | Yes | Yes | No | Yes | Yes | NA | Yes | NA | Yes | NA | Yes | No |
| 111. | Valencia2014 | Yes | Yes | Yes | Yes | No | Yes | Yes | No | Yes | NA | Yes | NA | Yes | No |
| 112. | Vasan1992 | Yes | Yes | Yes | Yes | No | Yes | Yes | No | Yes | NA | Yes | NA | Yes | No |
| 113. | Vitale1994 | Yes | Yes | Yes | Yes | NO | Yes | Yes | No | Yes | NA | Yes | NA | Yes | No |
| 114. | Witchitz1980 | Yes | Yes | Yes | Yes | No | Yes | Yes | No | Yes | NA | Yes | NA | Yes | No |

**References for Table 1:**

1.	Abbas S, Hameed N, Khilji SA, Jalal A. Outcome of thrombolysis with streptokinase in patients with prosthetic valve thrombosis. Pakistan journal of medical sciences. 2021;37(2):325-30.

2.	Agrawal D, Dubey S, Saket B, Bhargava M, Mehta N, Lohchab SS, et al. Thrombolytic therapy for prosthetic valve thrombosis in Third World countries. Indian heart journal. 1997;49(4):383-6.

3.	Ali A. Therapeutic Thrombolysis of Mechanical Prosthetic Valve Thrombosis Using Reteplase: An Effective and Safe Treatment Modality. EC Cardiology. 2021;8:75-81.

4.	Ahmed F, Shabir M. Management of Prosthetic Valve Thrombosis and its Clinical Profile in the Cardiac Center of Pakistan. Pakistan Journal of Medical and Health Sciences. 2022;16:1051-3.

5.	Ahn H, Kim KH, Kim KC, Kim CY. Surgical management of mechanical valve thrombosis: twenty-six years' experience. Journal of Korean medical science. 2008;23(3):378-82.

6.	Brik A, Salem A-M, Talat N, Shoukry A. Surgical Outcome of Prosthetic Heart Valve Obstruction: Single Center Experience. World Journal of Cardiovascular Surgery. 2012;Vol.02No.04:4.

7.	Ali A. Surgical outcomes of obstructive mechanical valve thrombosis, risk factors, and comorbidity analysis. Journal of Medicine in Scientific Research. 2019;2(4):303-.

8.	Aoyagi S, Fukunaga S, Suzuki S, Nishi Y, Oryoji A, Kosuga K. Obstruction of mechanical valve prostheses: clinical diagnosis and surgical or nonsurgical treatment. Surgery today. 1996;26(6):400-6.

9.	Azpitarte J, Sánchez-Ramos J, Urda T, Vivancos R, Oyonarte JM, Malpartida F. [Prosthetic valve thrombosis: which is the most appropriate initial therapy?]. Revista espanola de cardiologia. 2001;54(12):1367-76.

10.	Balram A, Kaul U, Rama Rao BV, Iyer KS, Rajani M, Rao IM, et al. Thrombotic obstruction of Bjork-Shiley valves--diagnostic and surgical considerations. International journal of cardiology. 1984;6(1):61-73.

11.	Bhatia S, Jain S, Sharma V, Mansuri Z, Patel K, Jain P, et al. Clinical profile of patients with prosthetic heart valve thrombosis undergoing fibrinolytic therapy and NYHA class as a predictor of outcome. Int J Cardiovasc Pract. 2021;6(1):e131484.

12.	Bollag L, Attenhofer Jost CH, Vogt PR, Linka AZ, Rickli H, Oechslin E, et al. Symptomatic mechanical heart valve thrombosis: high morbidity and mortality despite successful treatment options. Swiss medical weekly. 2001;131(9-10):109-16.

13.	Boukarroucha R, Massikh N, Chérif S, Lakehal R, Bendjaballah S, Aziza B, et al. Prosthetic Valve Thrombosis: About 205 Patients. Cardiovascular Diseases & Diagnosis. 2017;5(3):0-.

14.	Buttard P, Bonnefoy E, Chevalier P, Marcaz PB, Robin J, Obadia JF, et al. Mechanical cardiac valve thrombosis in patients in critical hemodynamic compromise. European journal of cardio-thoracic surgery : official journal of the European Association for Cardio-thoracic Surgery. 1997;11(4):710-3.

15.	Cáceres-Lóriga FM, Pérez-López H, Morlans-Hernández K, Facundo-Sánchez H, Santos-Gracia J, Valiente-Mustelier J, et al. Thrombolysis as first choice therapy in prosthetic heart valve thrombosis. A study of 68 patients. Journal of thrombosis and thrombolysis. 2006;21(2):185-90.

16.	Çakal B, Kalçık M, Güner A, Yesin M, Gürsoy MO, Gündüz S, et al. Improvement of endothelial function early after thrombolytic therapy in patients with prosthetic heart valve thrombosis. Turk Kardiyoloji Dernegi arsivi : Turk Kardiyoloji Derneginin yayin organidir. 2020;48(6):566-75.

17.	Copans H, Lakier JB, Kinsley RH, Colsen PR, Fritz VU, Barlow JB. Thrombosed Björk-Shiley mitral prostheses. Circulation. 1980;61(1):169-74.

18.	Karthikeyan G, Rajashekar P, Devasenapathy N, Biswas S, Kidambi B, Singal A, Mantoo MR, Soni M, Purohit G, Hote MP, Singh S, Velayoudam D. Urgent surgery vs fibrinolytic therapy for left-sided prosthetic valve thrombosis: a randomized trial. Eur Heart J. 2025 Sep 8;46(34):3373-3381. doi: 10.1093/eurheartj/ehaf391.

19.	Deviri E, Sareli P, Wisenbaugh T, Cronje SL. Obstruction of mechanical heart valve prostheses: clinical aspects and surgical management. Journal of the American College of Cardiology. 1991;17(3):646-50.

20.	Dürrleman N, Pellerin M, Bouchard D, Hébert Y, Cartier R, Perrault LP, et al. Prosthetic valve thrombosis: twenty-year experience at the Montreal Heart Institute. The Journal of thoracic and cardiovascular surgery. 2004;127(5):1388-92.

21.	Ermis N, Atalay H, Altay H, Bilgi M, Binici S, Sezgin AT. Comparison of fibrinolytic versus surgical therapy in the treatment of obstructive prosthetic valve thrombosis: a single-center experience. The heart surgery forum. 2011;14(2):E87-92.

22.	Farzaneh K, Mortazavi SH, Oraii A, Abbasi K, Salehi Omran A, Ahmadi Tafti SH, et al. Safety of thrombolytic therapy in patients with prosthetic heart valve thrombosis who have high international normalized ratio levels. Journal of cardiac surgery. 2020;35(10):2522-8.

23.	Guleria VS, Sharma P, Keshavamurthy G, Bharadwaj P, Datta R. Single-center Experience of Slow Thrombolysis in Stuck Mechanical Mitral Valve in India. Journal of Clinical and Preventive Cardiology. 2023;12(2):50-4.

24.	Gündüz S, Özkan M, Yesin M, Kalçık M, Gürsoy MO, Karakoyun S, et al. Prolonged Infusions of Low-Dose Thrombolytics in Elderly Patients With Prosthetic Heart Valve Thrombosis. Clinical and applied thrombosis/hemostasis : official journal of the International Academy of Clinical and Applied Thrombosis/Hemostasis. 2017;23(3):241-7.

25.	Gupta D, Kothari SS, Bahl VK, Goswami KC, Talwar KK, Manchanda SC, et al. Thrombolytic therapy for prosthetic valve thrombosis: short- and long-term results. American heart journal. 2000;140(6):906-16.

26.	Hirachan A, Roka M, Prajapati D, Adhikari C, kc B, Khadka M, et al. Prosthetic valve thrombosis in a tertiary cardiac centre. Nepalese Heart Journal. 2017;14:9-11.

27.	Huang F, Lan Y, Cheng Z, Zhang Z, Ren F. Thrombolytic treatment of prosthetic valve thrombosis: a study using Urokinase. Journal of cardiothoracic surgery. 2020;15(1):286.

28.	Issac KN. Prosthetic valve thrombosis and result study and thrombolysis outcomes. Journal of Cardiovascular Disease Research. 2023;14(1):3868-75.

29.	Kamal Y, Elshorbagy A, Mubarak Y, Elwany S, Shehata S. Thrombolysis Versus Redo-Surgery for Treatment of Mechanical Prosthetic Mitral Valve Thrombosis in Young Adult Patients. European Heart Journal Supplements. 2021;23.

30.	Karabulut MN, Günay R, Demirtaş MM. Obstructions of prosthetic heart valves: diagnosis and treatment considerations. The European Research Journal. 2024;10(4):361-70.

31.	Karthikeyan G, Math RS, Mathew N, Shankar B, Kalaivani M, Singh S, et al. Accelerated infusion of streptokinase for the treatment of left-sided prosthetic valve thrombosis: a randomized controlled trial. Circulation. 2009;120(12):1108-14.

32.	Karumuri S, Reddy HV, Kabra N. Prosthetic Valve Thrombosis: A Clinical, Echocardiographic and Cinefluoroscopic Study with Special Reference to Thrombolytic Therapy. International Journal of Science and Research (IJSR). 2019;8(11):233-7.

33.	Kathirvel D, Justin Paul G, Prathap Kumar G, Palanisamy G, Gnanavelu G, Ravishankar G, et al. Tenecteplase versus streptokinase thrombolytic therapy in patients with mitral prosthetic valve thrombosis. Indian heart journal. 2018;70(4):506-10.

34.	Katircioğlu SF, Ulus AT, Yamak B, Ozsöyler I, Birincioğlu L, Taşdemir O. Acute mechanical valve thrombosis of the St. Jude medical prosthesis. Journal of cardiac surgery. 1999;14(3):164-8.

35.	Keuleers S, Herijgers P, Herregods MC, Budts W, Dubois C, Meuris B, et al. Comparison of thrombolysis versus surgery as a first line therapy for prosthetic heart valve thrombosis. The American journal of cardiology. 2011;107(2):275-9.

36.	Khan A, Shah A, Shah S, Aslam M, Raza S. A Prospective Study on Treatment of Prosthetic Valve Thrombosis and its Clinical Presentation. Pakistan Journal of Medical and Health Sciences. 2021;16:871-3.

37.	Kiran GR, Chandrasekhar P, Mohammad Ali S. Clinical outcomes of patients with mitral prosthetic valve obstructive thrombosis treated with streptokinase or tenecteplase. Indian heart journal. 2021;73(3):365-8.

38.	Koca V, Bozat T, Sarikamis C, Akkaya V, Yavuz S, Ozdemir A. The use of transesophageal echocardiography guidance of thrombolytic therapy in prosthetic mitral valve thrombosis. The Journal of heart valve disease. 2000;9(3):374-8.

39.	Kontos GJ, Jr., Schaff HV, Orszulak TA, Puga FJ, Pluth JR, Danielson GK. Thrombotic obstruction of disc valves: clinical recognition and surgical management. The Annals of thoracic surgery. 1989;48(1):60-5.

40.	Kothari J, Patel K, Brahmbhatt B, Baria K, Talsaria M, Patel S, et al. Redo Mitral Valve Replacement for Prosthetic Valve Thrombosis: Single Center Experience. Journal of clinical and diagnostic research : JCDR. 2016;10(11):Pc01-pc3.

41.	Kumar S, Garg N, Tewari S, Kapoor A, Goel PK, Sinha N. Role of thrombolytic therapy for stuck prosthetic valves: a serial echocardiographic study. Indian heart journal. 2001;53(4):451-7.

42.	Kumar S, Lal S, Geelani M, Singh H, Metta V, Banik R. Effect of thrombolysis on stuck metallic valve prosthesis using Alteplase And Retplase. Indian Journal of Basic and Applied Medical Research. 2023; 12(4):48 – 53.

43.	Lafci B, Ozsöyler I, Kestelli M, Yilik L, Göktogan T, Karahan N, et al. Surgical treatment of prosthetic valve thrombosis: ten years' experience. The Journal of heart valve disease. 2006;15(3):400-3.

44.	Ledain LD, Ohayon JP, Colle JP, Lorient-Roudaut FM, Roudaut RP, Besse PM. Acute thrombotic obstruction with disc valve prostheses: diagnostic considerations and fibrinolytic treatment. Journal of the American College of Cardiology. 1986;7(4):743-51.

45.	Lengyel M, Vandor L. The role of thrombolysis in the management of left-sided prosthetic valve thrombosis: a study of 85 cases diagnosed by transesophageal echocardiography. The Journal of heart valve disease. 2001;10(5):636-49.

46.	López HP, Cáceres Lóriga FM, Hernàndez KM, Sánchez HF, González Jimenez N, Marrero Mirayaga MA, et al. Thrombolytic therapy with recombinant streptokinase for prosthetic valve thrombosis. Journal of cardiac surgery. 2002;17(5):387-93.

47.	Mahindru S, Pande S, Malhotra P, Thukral A, Kotwal AS, Gupta RP, et al. Mechanical prosthetic valve thrombosis in current era: 5-year follow-up. Indian journal of thoracic and cardiovascular surgery. 2021;37(2):140-6.

48.	Manandhar R, Prajapati D, Tamrakar R, Bogati A, Roka M, Sherpa K, et al. Clinical profile and management of prosthetic valve thrombosis in Tertiary cardiac Centre of Nepal, a prospective study. Nepalese Heart Journal. 2022;19:11-5.

49.	Mansuri Z, Sharma V, Jain S, Prajapati J, Bhatia S, Patel K. Clinical profile of prosthetic heart valve thrombosis and outcome analysis of fibrinolytic therapy versus surgical management: A single-center experience. Heart India. 2020;8:74.

50.	Manteiga R, Carlos Souto J, Altès A, Mateo J, Arís A, Dominguez JM, et al. Short-course thrombolysis as the first line of therapy for cardiac valve thrombosis. The Journal of thoracic and cardiovascular surgery. 1998;115(4):780-4.

51.	Maria Alberto Pomerantzeff P, Padovani Steffen S, Manuel de Almeida Brandão C, Aparecida Lapenna G, Tarasoutchi F, Biscegli Jatene F. Surgical Thrombectomy of Mechanical Valve Thrombosis. The Journal of heart valve disease. 2015;24(6):780-4.

52.	Martinell J, Jiménez A, Rábago G, Artiz V, Fraile J, Farré J. Mechanical cardiac valve thrombosis. Is thrombectomy justified? Circulation. 1991;84(5 Suppl):Iii70-5.

53.	Mashali MH, Ahmad Z, Omar Galal M, Al-Kouatli A. Effectiveness of alteplase infusion for the management of prosthetic mitral valve thrombosis in paediatric age group and proposed algorithm. Cardiology in the young. 2023;33(5):747-53.

54.	Milne O, Barthwal R, Agahari I, Ilton M, Kangaharan N. Management and Outcomes of Prosthetic Valve Thrombosis. An Australian Case Series From the Northern Territory. Heart, lung & circulation. 2020;29(3):469-74.

55.	Mishra C, Mishra A, Dash M, Routray S. Comparative safety and efficacy of tenecteplase and streptokinase in prosthetic valve thrombosis: An observation from Eastern India. Panacea Journal of Medical Sciences. 2022;12:538-44.

56.	Montorsi P, Cavoretto D, Alimento M, Muratori M, Pepi M. Prosthetic mitral valve thrombosis: can fluoroscopy predict the efficacy of thrombolytic treatment? Circulation. 2003;108 Suppl 1:Ii79-84.

57.	Mottahedi B, Kahrom M, Morovatdar N, Ghodsi M. Valve thrombectomy in Patients with Acute Prosthetic Valve Thrombosis; Long-term results. Archives of Pharmacy Practice. 2020;1:156.

58.	Nagy A, Dénes M, Lengyel M. Predictors of the outcome of thrombolytic therapy in prosthetic mitral valve thrombosis: a study of 62 events. The Journal of heart valve disease. 2009;18(3):268-75.

59.	Ali A, Naqvi SEH. Thrombolytic treatment of prosthetic valve thrombosis: a single center study using Tenectiplase. Indian Journal of Basic & Applied Medical Research. 2021;10(2).

60.	Narayan P, Prem A, Das D, Behera SK, Kumar N, Chowdhury SR, et al. Prosthetic valve thrombosis—should thrombolysis be considered as first line of management? Indian journal of thoracic and cardiovascular surgery. 2016;32(1):1-4.

61.	Nasri Nasrabadi A, Yarahmadi F, Amirpour A, Zavar R, Akbari M. Fibrinolytic Therapy in Thrombosis of Mechanical Valves: Outcomes and Complications. ARYA atherosclerosis. 2023;19(5):18-24.

62.	Nawale JM, Chaurasia AS, Nalawade DD, Abdagire N. Clinical Profile of Patients with Prosthetic Valve Thrombosis Treated with Fibrinolysis. Journal of the Practice of Cardiovascular Sciences. 2018;4(2):109-15.

63.	Nishanth KR, Shankar M, Srinivasa KH, Manjunath CN, Ravindranath KS. Fibrinolysis in left-sided mechanical prosthetic valve thrombosis with high INR. European heart journal Acute cardiovascular care. 2020;9(3_suppl):S58-s62.

64.	Ogutu P, Szalay Z, Roth M, Schönburg M, Kraus B, Klövekorn WP. Thrombectomy for prosthetic heart valve obstruction. Journal of cardiac surgery. 2007;22(3):218.

65.	Özkan M, Gündüz S, Biteker M, Astarcioglu MA, Çevik C, Kaynak E, et al. Comparison of different TEE-guided thrombolytic regimens for prosthetic valve thrombosis: the TROIA trial. JACC Cardiovascular imaging. 2013;6(2):206-16.

66.	Özkan M, Çakal B, Karakoyun S, Gürsoy OM, Çevik C, Kalçık M, et al. Thrombolytic therapy for the treatment of prosthetic heart valve thrombosis in pregnancy with low-dose, slow infusion of tissue-type plasminogen activator. Circulation. 2013;128(5):532-40.

67.	Özkan M, Gündüz S, Gürsoy OM, Karakoyun S, Astarcıoğlu MA, Kalçık M, et al. Ultraslow thrombolytic therapy: A novel strategy in the management of PROsthetic MEchanical valve Thrombosis and the prEdictors of outcomE: The Ultra-slow PROMETEE trial. American heart journal. 2015;170(2):409-18.

68.	Özkan M, Kalçık M, Gürsoy MO, Öcal L, Griffini S, Karakoyun S, et al. Assessment of Anti-Tissue Type Plasminogen Activator Antibodies in Patients With Prosthetic Heart Valve Thrombosis: The ATA Trial. Journal of cardiovascular pharmacology and therapeutics. 2016;21(4):372-80.

69.	Özkan M, Gündüz S, Güner A, Kalçık M, Gürsoy MO, Uygur B, et al. Thrombolysis or Surgery in Patients With Obstructive Mechanical Valve Thrombosis: The Multicenter HATTUSHA Study. Journal of the American College of Cardiology. 2022;79(10):977-89.

70.	Ozkokeli M, Sensoz Y, Ates M, Ekinci A, Akcar M, Yekeler I. Surgical treatment of left-sided prosthetic valve thrombosis: short and long-term results. International heart journal. 2005;46(1):105-11.

71.	Patil S, Setty N, Ramalingam R, Rudrappa MMB, Manjunath CN. Study of prosthetic heart valve thrombosis and outcomes after thrombolysis. Int J Res Med Sci. 2019;7(1):1074-8.

72.	Pavie A, Bors V, Baud F, Gandjbakhch I, Cabrol C. Surgery of prosthetic valve thrombosis. European heart journal. 1984;5 Suppl D:39-42.

73.	Potwar S, Kothari A, Potnis A, Paranjape M. Thrombolytic therapy as first line treatment in prosthetic mitral valve thrombosis. International Surgery Journal. 2023;10(6):1019-23.

74.	Pradhan A, Bhandari M, Gupta V, Vishwakarma P, Sethi R, Narain VS, et al. Short-Term Clinical Follow-Up After Thrombolytic Therapy in Patients With Prosthetic Valve Thrombosis: A Single-Center Experience. Cardiology research. 2019;10(6):345-9.

75.	S P, K S, Krishnappa S, S R, N M. Efficacy and safety of thrombolytic therapy in prosthetic valve thrombosis. International Journal of Research in Medical Sciences. 2020;8:575.

76.	Raboi A, Al-Motarreb A, Al-Kanadi A, Abdulmughni AA, Kadi A. Mechanical valve dysfunction in yemen. Heart views : the official journal of the Gulf Heart Association. 2010;11(2):47-51.

77.	Rajasekhar D, Balakrishnan KG, Venkitachalam CG, Tharakan JA, Titus T, Pillai VR, et al. Thrombolytic therapy for prosthetic cardiac valve thrombosis. Indian heart journal. 1994;46(2):101-5.

78.	Raman K, Mohanraj A, Palanisamy V, Ganesh J, Rawal S, Kurian VM, et al. Thrombolysis and surgery for mitral prosthetic valve thrombosis: 11-year outcomes. Asian cardiovascular & thoracic annals. 2019;27(8):633-40.

79.	Ramos AI, Ramos RF, Togna DJ, Arnoni AS, Staico R, Galo MM, et al. Fibrinolytic therapy for thrombosis in cardiac valvular prosthesis short and long term results. Arquivos brasileiros de cardiologia. 2003;81(4):393-8, 87-92.

80.	Rao S, Sreedhara R, Upadhya KG, Basavanagoud N, Manjunath C. CLINICAL PROFILE AND OUTCOME OF PROSTHETIC VALVE THROMBOSIS PATIENTS TREATED WITH THROMBOLYSIS. Journal of Cardiovascular Disease Research. 2023;14(09).

81.	Rath DP, Kumar S, Pai S, Padmanabhan R. A retrospective study of outcome of prosthetic heart valve occlusion cases at JIPMER. IOSR-JDMS. 2019;18:68-71.

82.	Reddy NK, Padmanabhan TN, Singh S, Kumar DN, Raju PR, Satyanarayana PV, et al. Thrombolysis in left-sided prosthetic valve occlusion: immediate and follow-up results. The Annals of thoracic surgery. 1994;58(2):462-70; discussion 70-1.

83.	Renzulli A, Onorati F, De Feo M, Vitale N, Esposito S, Agozzino L, et al. Mechanical valve thrombosis: a tailored approach for a multiplex disease. The Journal of heart valve disease. 2004;13 Suppl 1:S37-42.

84.	Careaga-Reyna G, Esparza-Pantoja J, Argüero-Sánchez R. [Thrombosis of mechanical heart valve prosthesis. Predisposing factors and results of surgical treatment]. Gaceta medica de Mexico. 1997;133(6):535-9.

85.	Roudaut R, Labbe T, Lorient-Roudaut MF, Gosse P, Baudet E, Fontan F, et al. Mechanical cardiac valve thrombosis. Is fibrinolysis justified? Circulation. 1992;86(5 Suppl):Ii8-15.

86.	Roudaut R, Lafitte S, Roudaut M-F, Reant P, Pillois X, Durrieu-Jaïs C, et al. Management of prosthetic heart valve obstruction: Fibrinolysis versus surgery. Early results and long-term follow-up in a single-centre study of 263 cases. Archives of Cardiovascular Diseases. 2009;102(4):269-77.

87.	Sadeghipour P, Saedi S, Saneei L, Rafiee F, Yoosefi S, Parsaee M, et al. Fast vs. ultraslow thrombolytic infusion regimens in patients with obstructive mechanical prosthetic valve thrombosis: a pilot randomized clinical trial. European heart journal Cardiovascular pharmacotherapy. 2022;8(7):668-76.

88.	Sadeghipour P, Salehi M, Kohansal E, Mozafarybazargany M, Parsaee M, Farrashi M, et al. Ultraslow low-dose thrombolytic therapy in patients with acute mechanical prosthetic heart valve thrombosis &#x2013; A prospective cohort study. International journal of cardiology. 2024;416.

89.	Sakr SA, Abdel-Shakour H, Ramadan MM. Successful fibrinolytic treatment of prosthetic heart valve thrombosis using streptokinase. Acta clinica Belgica. 2015;70(3):159-66.

90.	Saleh H, Azzam M, Swailum AA, Farouk A. Early Outcomes of Emergency Surgery for Left-Sided Mechanical Valve Thrombosis. The heart surgery forum. 2021;24(6):E983-e7.

91.	Santos H, González-Pacheco H, Ruan L, Espinosa J, Gopar-Nieto R, Manzur-Sandoval D, et al. THROMBOLYTIC THERAPY COMPARED WITH URGENT SURGERY IN THE TREATMENT OF PROSTHETIC VALVE THROMBOSIS. A 15 YEARS EXPERIENCE IN A SINGLE CENTER. Journal of the American College of Cardiology. 2021;77:1729.

92.	Separham A, Ghaffari S, Aslanabadi N, Sohrabi B, Ghojazadeh M, Anamzadeh E, et al. Prosthetic valve thrombosis. Journal of cardiac surgery. 2015;30(3):246-50.

93.	Shapira Y, Herz I, Vaturi M, Porter A, Adler Y, Birnbaum Y, et al. Thrombolysis is an effective and safe therapy in stuck bileaflet mitral valves in the absence of high-risk thrombi. Journal of the American College of Cardiology. 2000;35(7):1874-80.

94.	Sharif Khan H, Ijaz Z, Ali M, Saif M, Ishaq U, Kamal A, et al. Clinical Outcomes of Mechanical Prosthetic Valve Thrombosis. Cureus. 2020;12(6):e8760.

95.	Sharma V, K. Arora BY, Gupta LC, Poonia A, Raina S, Yadav US, et al. The Efficacy and Safety of Thrombolytic Agents for Patients with Prosthetic Valve Thrombosis. Journal of the Practice of Cardiovascular Sciences. 2022;8(1):30-4.

96.	Sharma V, Singh R, Mishra R, Arora A, Gupta L, Yadava OP. Use of tenecteplase for left-sided prosthetic valve thrombosis. The Journal of the Association of Physicians of India. 2012;60:55-8.

97.	Shojaeifard M, Koochari I, Zarei K, Mohammadabadi HD, Erami S, Khoshtinat V, et al. Evaluation of Response to Thrombolytic Therapy versus Surgery in Patients with Aortic Prosthetic Valve Thrombosis in a Tertiary Heart Center in Tehran, Iran, during 2007–2019. Research in Cardiovascular Medicine. 2023;12(1):33-8.

98.	Showkathali R, Yalamanchi R, Oomman A, Abhinaya K. Thrombolysis in high-risk patients with left-sided obstructive prosthetic valve thrombosis. Kardiologia polska. 2020;78:1166-8.

99.	Silber H, Khan SS, Matloff JM, Chaux A, DeRobertis M, Gray R. The St. Jude valve. Thrombolysis as the first line of therapy for cardiac valve thrombosis. Circulation. 1993;87(1):30-7.

100.	Singh AK, Agarwal S, Satyarthi S, Kunal, Satsangi DK. Comparison of thrombolytic therapy for prosthetic valve thrombosis at the mitral and aortic position. Indian journal of thoracic and cardiovascular surgery. 2011;27(3):114-8.

101.	Singh S, Doshi S, Salahuddin S, Tarik M, Barwad P, Ramakrishnan L, et al. Antistreptokinase antibodies and outcome of fibrinolytic therapy with streptokinase for left-sided prosthetic valve thrombosis. American heart journal. 2015;169(1):170-4.

102.	Solorio S, Sánchez H, Madrid R, Badui E, Valdespino A, Murillo H, et al. [Thrombolysis in mechanical prosthetic valve thrombosis. Its management with streptokinase]. Archivos del Instituto de Cardiologia de Mexico. 1994;64(1):51-5.

103.	Tadesse KD, Gebru M, Tekleab AM. Treatment outcome of patients with prosthetic stuck valves at the Cardiac Center of Ethiopia. PloS one. 2023;18(4):e0284652.

104.	Taljaard JJ, Doubell AF. Prosthetic valve obstruction at Tygerberg Hospital between January 1991 and February 2001. Cardiovascular journal of South Africa : official journal for Southern Africa Cardiac Society [and] South African Society of Cardiac Practitioners. 2003;14(4):182-8.

105.	Tareen H, Saad M, Bashir M, Memon T, Hussain I, Khan M. Clinical Profile and Management of Prosthetic Valve Thrombosis in Tertiary Cardiac Centre. Pakistan Journal of Medical and Health Sciences. 2022;16:623-5.

106.	Teshima H, Hayashida N, Nishimi M, Tayama E, Fukunaga S, Tomoeda H, et al. Thrombolytic therapy with tissue plasminogen activator for the treatment of nonstructural malfunction of bileaflet cardiac valve prostheses. Artificial organs. 2002;26(5):460-6.

107.	Toker ME, Eren E, Balkanay M, Kirali K, Yanartaş M, Calişkan A, et al. Multivariate analysis for operative mortality in obstructive prosthetic valve dysfunction due to pannus and thrombus formation. International heart journal. 2006;47(2):237-45.

108.	Tong AT, Roudaut R, Ozkan M, Sagie A, Shahid MS, Pontes Júnior SC, et al. Transesophageal echocardiography improves risk assessment of thrombolysis of prosthetic valve thrombosis: results of the international PRO-TEE registry. Journal of the American College of Cardiology. 2004;43(1):77-84.

109.	Tsai KT, Lin PJ, Chang CH, Chu JJ, Chang JP, Kao CL, et al. Surgical management of thrombotic disc valve. The Annals of thoracic surgery. 1993;55(1):98-101.

110.	Valencia Sánchez JS, Arriaga Nava R, Martínez Enríquez A, Navarro Robles J, Palomo Villada JA. [Medical-surgical management of left heart valve prosthesis dysfunction due to obstruction. Eleven year's experience]. Archivos del Instituto de Cardiologia de Mexico. 1999;69(2):127-33.

111.	Valencia-Sánchez JS, Arriaga-Nava R. [Thrombolysis in left mechanical prosthetic heart valve obstruction by thrombus. Following and results]. Revista medica del Instituto Mexicano del Seguro Social. 2014;52(6):684-91.

112.	Vasan RS, Kaul U, Sanghvi S, Kamlakar T, Negi PC, Shrivastava S, et al. Thrombolytic therapy for prosthetic valve thrombosis: a study based on serial Doppler echocardiographic evaluation. American heart journal. 1992;123(6):1575-80.

113.	Vitale N, Renzulli A, Cerasuolo F, Caruso A, Festa M, de Luca L, et al. Prosthetic valve obstruction: thrombolysis versus operation. The Annals of thoracic surgery. 1994;57(2):365-70.

114.	Witchitz S, Veyrat C, Moisson P, Scheinman N, Rozenstajn L. Fibrinolytic treatment of thrombus on prosthetic heart valves. British heart journal. 1980;44(5):545-54.
