# Supplementary material for: Thrombolytic therapy versus cardiac surgery for left-sided prosthetic heart valve thrombosis: A systematic review and meta-analysis
Source: Am Heart J Plus. 2026 Jul 29;69:100849. doi: 10.1016/j.ahjo.2026.100849 (PMC13451796; doi:10.1016/j.ahjo.2026.100849)
Supplement: Supplementary file 2 [file mmc2.docx]

Galbraith plot: Heterogeneity evaluation for success in fibrinolytic treatment

Figure 1. Galbraith plot for Heterogeneity evaluation for success in fibrinolytic treatment

| Table 1. Heterogeneity evaluation for success in fibrinolytic treatment | | | | | | | |
| --- | --- | --- | --- | --- | --- | --- | --- |
| Pooled rate (95%CI) | $\boldsymbol{\tau}^{\mathbf{2}}$ | $\mathbf{I}^{\mathbf{2}}$ | **Chi2** | | | **Result** | **Removed Study** |
|  |  |  | **Q** | **df** | **P-Value** |  |  |
| 0.837  (0.815 – 0.857) | 0.2824 | 62.7 % | 224.98 | 84 | <0.0001 | Heterogen | **Without removed** |
| 0.840  (0.818 – 0.859) | 0.2568 | 58.1 % | 198.11 | 83 | <0.0001 | Heterogen | **Karthikeyan 2009** |
| 0.842  (0.821 – 0.861) | 0.2275 | 55.7 % | 185.25 | 82 | <0.0001 | Heterogen | **Renzulli 2004** |
| 0.839  (0.818 – 0.857) | 0.2043 | 53.6 % | 174.42 | 81 | <0.0001 | Heterogen | **Sadeghipour 2022** |
| 0.841  (0.821 – 0.859 | 0.1804 | 51.2 % | 163.98 | 80 | <0.0001 | Heterogen | **Valencia 20140** |
| 0.843  (0.824 – 0.861) | 0.1685 | 48.2 % | 152.60 | 79 | <0.0001 | Heterogen | **Roudaut 20090** |
| 0.845  (0.826 – 0.862) | 0.1486 | 45.1 % | 141.98 | 78 | <0.0001 | Heterogen | **Karumuri 20190** |
| 0.843  (0.824 – 0.860) | 0.1331 | 43.1 % | 135.30 | 77 | <0.0001 | Heterogen | **Nasrabadi 2023** |
| 0.840  (0.822 – 0.857) | 0.1211 | 41.4 % | 129.74 | 76 | 0.0001 | Heterogen | **Sharma 2022** |
| 0.838  (0.820 – 0.855) | 0.1062 | 38 % | 121.01 | 75 | 0.0006 | Heterogen | **Çakal 2020** |
| 0.836  (0.818 – 0.853) | 0.0983 | 35.9 % | 115.47 | 74 | 0.0015 | Heterogen | **Purushotama 2020** |
| 0.834  (0.816 – 0.850) | 0.0839 | 32.4 % | 107.97 | 73 | 0.0049 | Heterogen | **Kiran 2021** |
| 0.836  (0.818 – 0.852) | 0.0731 | 28.6 % | 100.89 | 72 | 0.014 | Heterogen | **Separham 2015** |
| 0.837  (0.820 – 0.853) | 0.0669 | 24.8 % | 94.47 | 71 | 0.033 | Heterogen | **Aoyagi 1996** |
| 0.839  (0.823 – 0.855) | 0.0574 | 21.3 % | 88.95 | 70 | 0.063 | Homogen | **Abbas 2020** |

Funnel Plot: Publication bias evaluation for success in fibrinolytic treatment

Figure 2. Funnel Plot for Publication bias evaluation for success in fibrinolytic treatment

| Table 2. Publication bias evaluation for success in fibrinolytic treatment | | | | | | | | | | | | | | | | |
| --- | --- | --- | --- | --- | --- | --- | --- | --- | --- | --- | --- | --- | --- | --- | --- | --- |
| Begg | | | | **Egger** | | | | | **Publication bias** | **Pooled rate (95%CI)** | $\boldsymbol{\tau}^{\mathbf{2}}$ | $\mathbf{I}^{\mathbf{2}}$ | **Chi2** | | |  |
| Z | **P-Value** | **Bias estimate** | **SE** | **t** | **Df** | **P-Value** | **Bias estimate** | **SE** |  |  |  |  | **Q** | **df** | **P-Value** | **Removed study** |
| 2.06 | 0.040 | 414 | 201.45 | 2.83 | 69 | 0.006 | 0.8785 | 0.311 | Was seen | 0.839  (0.823 – 0.855) | 0.0574 | 21.3 % | 88.95 | 70 | 0.063 | **Without removed** |
| 1.77 | 0.076 | 350 | 197.24 | 2.55 | 68 | 0.013 | 0.8113 | 0.3178 | Wae not seen. | 0.839  (0.822 – 0.854) | 0.0564 | 19.8 % | 86.08 | 69 | 0.080 | **Özkan 2013b** |

Galbraith Plot: Heterogeneity evaluation for success in surgery treatment

Figure 3. Galbraith Plot Heterogeneity evaluation for success in surgery treatment

| Table 3. Heterogeneity evaluation for success in surgery treatment | | | | | | | |
| --- | --- | --- | --- | --- | --- | --- | --- |
| Pooled rate (95%CI) | $\tau^{2}$ | $I^{2}$ | **Chi2** | | | **Result** | **Removed Study** |
|  |  |  | **Q** | **df** | **P-Value** |  |  |
| 0.835  (0.799 – 0.868) | 0.4082 | 67.6 % | 138.68 | 45 | <0.0001 | Heterogen | **Without removed** |
| 0.840  (0.808 – 0.867) | 0.2914 | 62.8 % | 118.19 | 44 | <0.0001 | Heterogen | **Keuleers 20110** |
| 0.843  (0.814 – 0.868) | 0.2129 | 57.9 % | 102.15 | 43 | <0.0001 | Heterogen | **Taliaard 20030.** |
| 0.846  (0.820 – 0.870) | 0.1616 | 49.5 % | 83.21 | 42 | 0.0002 | Heterogen | **Saleh 2021** |
| 0.850  (0.830 – 0.871) | 0.1005 | 42.4 % | 71.21 | 41 | 0.002 | Heterogen | **Pavie 1984** |
| 0.854  (0.834 – 0.871 | 0.0344 | 34.8 % | 61.33 | 40 | 0.017 | Heterogen | **Kothari 20160** |
| 0.851  (0.833 – 0.867) | 0.0101 | 27.2 % | 53.56 | 39 | 0.060 | Homogen | **Alaa 2012** |

Funnel Plot: Publication bias evaluation for success in surgery treatment

Figure 4. Funnel Plot for Publication bias evaluation for success in surgery treatment

| Table 4. Publication bias evaluation for success in surgery treatment | | | | | | | | | | | | | | | | |
| --- | --- | --- | --- | --- | --- | --- | --- | --- | --- | --- | --- | --- | --- | --- | --- | --- |
| Begg | | | | **Egger** | | | | | **Publication bias** | **Pooled rate (95%CI)** | $\boldsymbol{\tau}^{\mathbf{2}}$ | $\mathbf{I}^{\mathbf{2}}$ | **Chi2** | | |  |
| Z | **P-Value** | **Bias estimate** | **SE** | **t** | **Df** | **P-Value** | **Bias estimate** | **SE** |  |  |  |  | **Q** | **df** | **P-Value** | **Removed study** |
| 2.16 | 0.031 | 185 | 85.76 | 1.65 | 38 | 0.108 | 0.5821 | 0.3532 | Was not seen. | 0.851  (0.833 – 0.867) | 0.0101 | 27.2 % | 53.56 | 39 | 0.060 | **Without removed** |

Galbraith Plot: Heterogeneity evaluation for mortality in fibrinolytic treatment

Figure 5. Galbraith Plot: Heterogeneity evaluation for mortality in fibrinolytic treatment

| Table 5. Heterogeneity evaluation for mortality in fibrinolytic treatment | | | | | | | |
| --- | --- | --- | --- | --- | --- | --- | --- |
| Pooled rate (95%CI) | $\boldsymbol{\tau}^{\mathbf{2}}$ | $\mathbf{I}^{\mathbf{2}}$ | **Chi2** | | | **Result** | **Removed study** |
|  |  |  | **Q** | **df** | **P-Value** |  |  |
| 0.072  (0.061 – 0.086) | 0.2785 | 42.8 % | 146.93 | 84 | <0.0001 | Heterogen | **Without removed** |
| 0.071  (0.060 – 0.084) | 0.2346 | 36.5 % | 130.78 | 83 | 0.0006 | Heterogen | **Abbas 2020** |
| 0.070  (0.060 – 0.082) | 0.1451 | 23.2 % | 106.80 | 82 | 0.034 | Heterogen | **Karumuri 2019** |
| .0734  (0.063 – 0.085) | 0.1066 | 18.6 % | 99.56 | 81 | 0.079 | Homogen | **Özkan 20130** |

Funnel Plot: Publication bias evaluation for mortality in fibrinolytic treatment

Figure 6. Funnel Plot Publication bias evaluation for mortality in fibrinolytic treatment

| Table 6. Publication bias evaluation for mortality in fibrinolytic treatment | | | | | | | | | | | | | | | | |
| --- | --- | --- | --- | --- | --- | --- | --- | --- | --- | --- | --- | --- | --- | --- | --- | --- |
| Begg | | | | **Egger** | | | | | **Publication bias** | **Pooled rate (95%CI)** | $\boldsymbol{\tau}^{\mathbf{2}}$ | $\mathbf{I}^{\mathbf{2}}$ | **Chi2** | | |  |
| Z | **P-Value** | **Bias estimate** | **SE** | **t** | **Df** | **P-Value** | **Bias estimate** | **SE** |  |  |  |  | **Q** | **df** | **P-Value** | **Removed study** |
| -3.28 | 0.001 | -820 | 249.63 | -7.33 | 80 | <0.0001 | -1.5267 | 0.208 | Was seen | 0.0734  (0.063 – 0.085) | 0.1066 | 18.6 % | 99.56 | 81 | 0.079 | **Without removed** |
| -3.18 | 0.0015 | -799 | 245.11 | -7.09 | 79 | <0.0001 | -1.4914 | 0.210 | Was seen. | 0.074  (0.064 – 0.086) | 0.099 | 16.6 % | 95.94 | 80 | 0.108 | **Çakal 2020** |
| -3.08 | 0.002 | -741 | 240.60 | -6.87 | 78 | <0.0001 | -1.4614 | 0.213 | Was seen. | 0.075  (0.065 – 0.087) | 0.0934 | 15 % | 92.99 | 79 | 0.134 | **Farzaneh 2020** |
| -3.01 | 0.003 | -710 | 236.13 | -6.66 | 77 | <0.0001 | -1.4404 | 0.216 | Was seen. | 0.076  (0.065 – 0.088) | 0.0894 | 14.2 % | 90.90 | 78 | 0.151 | **Reddy 1994** |
| -2.94 | 0.0032 | -682 | 231.68 | -6.46 | 76 | <0.0001 | -1.4210 | 0.220 | Was seen. | 0.0762  (0.065 – 0.088) | 0.0858 | 13.5 % | 88.99 | 77 | 0.165 | **Naqvi 2021** |
| -2.91 | 0.004 | -661 | 227.26 | -6.32 | 75 | <0.0001 | -1.3665 | 0.216 | Was seen. | 0.078  (0.067 – 0.09) | 0.070 | 8.3 % | 82.89 | 76 | 0.275 | **Sadeghipour 2024** |
| -2.84 | 0.0046 | -632 | 222.88 | -6.15 | 74 | <0.0001 | -1.3545 | 0.2204 | Was seen | 0.078  (0.068 – 0.09) | 0.0682 | 8.1 % | 81.58 | 75 | 0.282 | **Özkan 2013b** |
| -2.77 | 0.0056 | -605 | 218.52 | -5.97 | 73 | <0.0001 | -1.3417 | 0.2246 | Was seen. | 0.079  (0.068 – 0.091) | 0.0661 | 7.8 % | 80.27 | 74 | 0.289 | **Ozkan 2016** |
| -2.71 | 0.007 | -580 | 214.19 | -5.80 | 72 | <0.0001 | -1.2850 | 0.2214 | Was seen. | 0.080  (0.070 – 0.092) | 0.0538 | 2.2 % | 74.66 | 73 | 0.424 | **Özkan 2015** |
| -2.62 | 0.009 | -594 | 209.89 | -5.64 | 71 | <0.0001 | -1.2259 | 0.2175 | Was seen. | 0.082  (0.071 – 0.094) | 0.0427 | 0 % | 68.99 | 72 | 0.579 | **Sadeghipour 2022** |
| -2.53 | 0.011 | -521 | 205.61 | -5.45 | 70 | <0.0001 | -1.2092 | 0.2219 | Was seen. | 0.082  (0.071 – 0.094) | 0.0411 | 0 % | 67.70 | 71 | 0.589 | **Teshima 2002** |
| -2.39 | 0.017 | -482 | 201.37 | -5.27 | 69 | <0.0001 | -1.1926 | 0.2265 | Was seen. | 0.083  (0.072 – 0.095) | 0.0396 | 0 % | 66.45 | 70 | 0.5981 | **Renzulli 2004** |
| -2.31 | 0.021 | -456 | 197.15 | -5.11 | 68 | <0.0001 | -1.1822 | 0.2315 | Was seen. | 0.083  (0.072 – 0.095) | 0.0385 | 0 % | 65.51 | 69 | 0.5968 | **Koca 2000** |
| -2.22 | 0.026 | -429 | 192.97 | -4.59 | 67 | <0.0001 | -1.1728 | 0.2369 | Was seen. | 0.083  (0.073 – 0.095) | 0.0375 | 0 % | 64.63 | 68 | 0.5934 | **Mashali 2023** |
| -2.15 | 0.032 | -405 | 188.81 | -4.80 | 66 | <0.0001 | -1.1646 | 0.2426 | Was seen. | 0.084  (0.073 – 0.096) | 0.0366 | 0 % | 63.81 | 67 | 0.5878 | **Azpitarate 2001** |
| -2.09 | 0.037 | -386 | 184.68 | -4.67 | 65 | <0.0001 | -1.1600 | 0.2486 | Was seen. | 0.084  (0.073 – 0.096) | 0.0359 | 0 % | 63.13 | 66 | 0.5776 | **Montorsi 2003** |
| -2.04 | 0.042 | -368 | 180.59 | -4.54 | 64 | <0.0001 | -1.1575 | 0.2551 | Was seen. | 0.084  (0.073 – 0.096) | 0.0353 | 0 % | 62.50 | 65 | 0.5649 | **Vasan 1992** |
| -2.02 | 0.043 | -357 | 176.52 | -4.44 | 63 | <0.0001 | -1.1631 | 0.2619 | Was seen. | 0.084  (0.074 – 0.097) | 0.0350 | 0 % | 62.08 | 64 | 0.5448 | **Keuleers 2011** |
| -2.02 | 0.043 | -349 | 172.49 | -4.37 | 62 | <0.0001 | -1.1763 | 0.2690 | Was seen. | 0.085  (0.074 – 0.097) | 0.0349 | 0 % | 61.79 | 63 | 0.5195 | **Tadesse 2023** |
| -1.95 | 0.052 | -328 | 168.49 | -4.16 | 61 | 0.0001 | -1.1290 | 0.2716 | Was not seen | 0.085  (0.075 – 0.098) | 0.0308 | 0 % | 59.35 | 62 | 0.5721 | **Kathirvel 2018** |

Galbraith Plot: Heterogeneity evaluation for mortality in surgery treatment

Figure 7. Galbraith Plot: Heterogeneity evaluation for mortality in surgery treatment

| Table 7. Heterogeneity evaluation for mortality in surgery treatment | | | | | | | |
| --- | --- | --- | --- | --- | --- | --- | --- |
| Pooled rate (95%CI) | $\boldsymbol{\tau}^{\mathbf{2}}$ | $\mathbf{I}^{\mathbf{2}}$ | **Chi2** | | | **Result** | **Removed study** |
|  |  |  | **Q** | **df** | **P-Value** |  |  |
| 0.162  (0.135 – 0.193) | 0.2850 | 62.1 % | 118.63 | 45 | <0.0001 | Heterogen | **Without removed study** |
| 0.158  (0.132 – 0.188 | 0.2530 | 56.4 % | 101 | 44 | <0.0001 | Heterogen | **Saleh 2021** |
| 0.154  (0.131 – 0. 180) | 0.1629 | 48.7 % | 83.87 | 43 | 0.0002 | Heterogen | **Taliaard 2003** |
| 0.151  (0.130 – 0.174) | 0.1084 | 41.6 % | 71.86 | 42 | 0.003 | Heterogen | **Pavie 1984** |
| 0.155  (0.135 – 0.178) | 0.0725 | 35.6 % | 63.64 | 41 | 0.013 | Heterogen | **Alaa 2012** |
| 0.150  (0.134 – 0.168) | 0.0166 | 26.3 % | 54.27 | 40 | 0.066 | Homogen | **Kothari 2016** |

Funnel Plot: Publication bias evaluation for mortality in surgery treatment

Figure 8. Funnel Plot Publication bias evaluation for mortality in surgery treatment

| Table 8. Publication bias evaluation for mortality in surgery treatment | | | | | | | | | | | | | | | | |
| --- | --- | --- | --- | --- | --- | --- | --- | --- | --- | --- | --- | --- | --- | --- | --- | --- |
| Begg | | | | **Egger** | | | | | **Publication bias** | **Pooled rate (95%CI)** | $\boldsymbol{\tau}^{\mathbf{2}}$ | $\mathbf{I}^{\mathbf{2}}$ | **Chi2** | | |  |
| Z | **P-Value** | **Bias estimate** | **SE** | **t** | **Df** | **P-Value** | **Bias estimate** | **SE** |  |  |  |  | **Q** | **df** | **P-Value** | **Removed study** |
| -2.05 | 0.041 | -182 | 88.99 | -1.41 | 39 | 0.165 | -0.5019 | 0.3551 | Was not seen. | 0.150  (0.134 – 0.168) | 0.0166 | 26.3 % | 54.27 | 40 | 0.066 | With out Removed study |
